# Decoupling Josephson Coupling and Supercurrent Nonreciprocity in Twisted NbSe_2_/NbSe_2_ van der Waals Junctions

**DOI:** 10.1021/acs.nanolett.6c03685

**Published:** 2026-10-23

**Authors:** Seung-Gi Lee, Kwon-Neong Jung, Jae-Keun Kim, Jae-Chun Jeon, Jiho Yoon, Stuart S. P. Parkin, Kun-Rok Jeon

**Affiliations:** † Department of Physics, Chung-Ang University (CAU), Seoul 06974, Republic of Korea; ‡ Max Planck Institute of Microstructure Physics, Weinberg 2, 06120 Halle (Saale), Germany; § Department of Physics, 35010 University of Seoul, Seoul 02504, Republic of Korea

**Keywords:** Josephson coupling, supercurrent nonreciprocity, van der Waals superconductors, twisted NbSe_2_/NbSe_2_ junctions, interface disorder

## Abstract

The microscopic origin of supercurrent nonreciprocity in van der Waals Josephson junctions remains under debate, particularly regarding the role of twist-angle engineering. Here, we investigate twisted NbSe_2_/NbSe_2_ vertical Josephson junctions with controlled crystallographic alignment and clean interfaces. High-resolution transmission electron microscopy directly determines the twist angle and interface quality. While Josephson coupling exhibits a pronounced twist-angle dependence, the supercurrent diode efficiency remains small and shows no systematic angular dependence. In contrast, enhanced diode responses emerge in weakly coupled junctions with reduced transparency and interfacial disorder. Deliberate interface degradation further enhances the diode efficiency to nearly 30% and produces irregular magnetic-field dependence. These results demonstrate a clear decoupling between Josephson coupling and supercurrent nonreciprocity, indicating that interfacial disorder rather than twist-angle-controlled momentum matching plays a dominant role in the observed diode response. Our findings establish an important benchmark for understanding nonreciprocal superconducting transport in van der Waals Josephson devices in the future.

## Graphical abstract

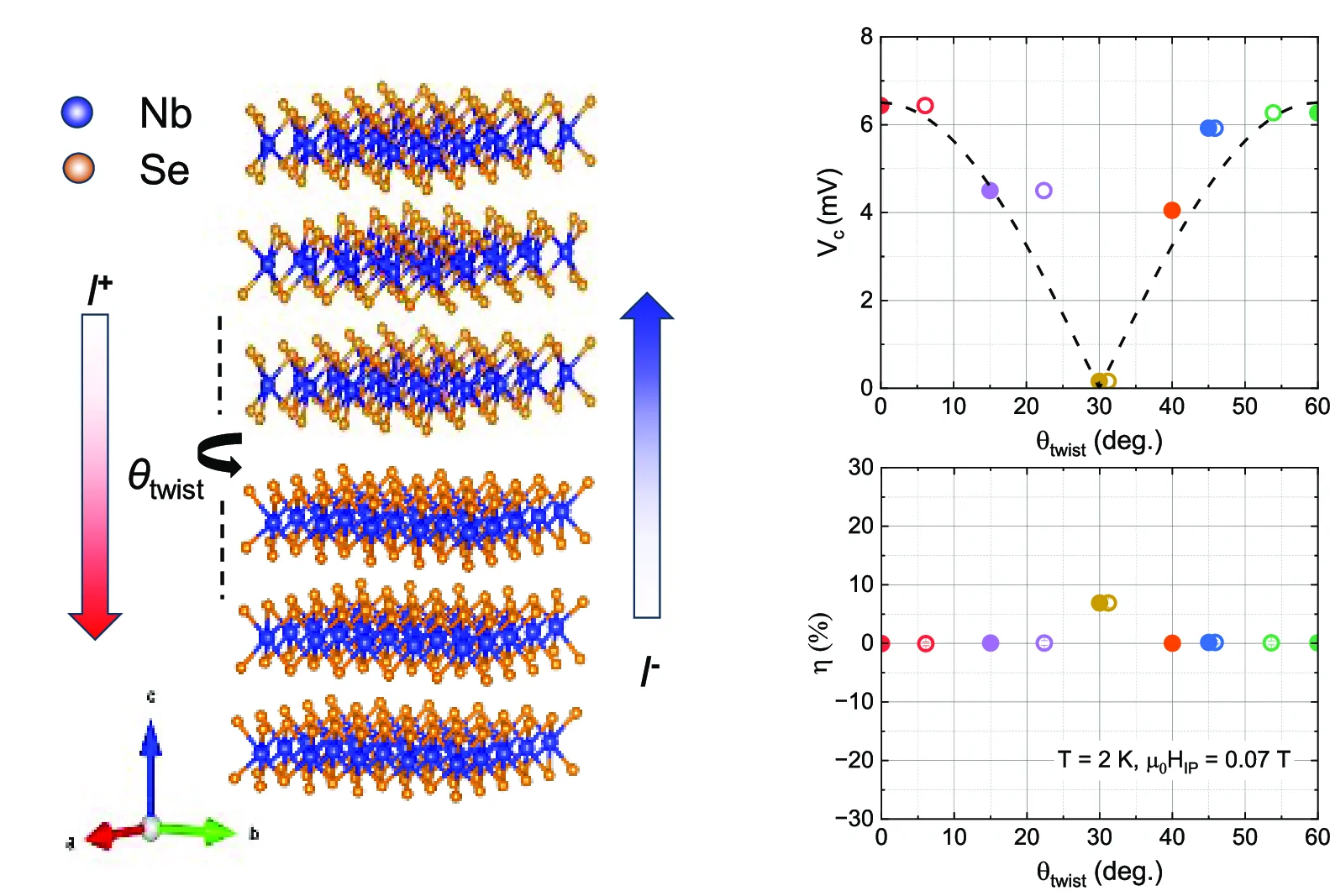

The van der Waals (vdW) vertical Josephson junctions (JJs) provide a powerful platform for exploring superconducting transport in atomically engineered interfaces, enabling both device applications and the study of emergent quantum phenomena.^1–8^ These structures, typically assembled from two-dimensional (2D) superconductors, offer clean and highly controllable interfaces in which electronic coupling can be tuned with high precision.^9–11^ Among the available tuning parameters, the crystallographic twist angle θ_twist_ between stacked layers has emerged as a central degree of freedom for modulating Josephson transport.^2,6,7,12^ In layered superconductors such as 2H-NbSe_2_, which is known to exhibit strong Ising superconductivity and enhanced in-plane field robustness in the atomic limit,^13,14^ twist-angle engineering is widely understood to influence Cooper-pair tunneling through momentum-space matching of the superconducting electrodes.^2,6,7,12^ Because the relative alignment of Fermi surfaces depends on θ_twist_, the Josephson critical current is expected to exhibit characteristic angular periodicity, with enhanced coupling near crystallographically equivalent orientations (θ_twist_ = 0°, 60°, 120°, ...) and suppression at intermediate angles.^2,6,7,12^ This behavior has been interpreted as evidence that twist-controlled momentum conservation governs interlayer superconducting transport in vdW JJs.

More recently, vdW Josephson systems have also been explored in the context of the Josephson diode effect (JDE), where the critical current becomes asymmetric under magnetic field reversal, *I*_c_^+^ ≠ |*I*_c_^–^|.^4,15,16^ The diode efficiency, $\eta =\frac{I_{c}^{+}-\left|I_{c}^{-}\right|}{I_{c}^{+}+\left|I_{c}^{-}\right|}$ , is often used to quantify this nonreciprocity. Several theoretical and experimental studies have suggested that symmetry breaking associated with twist angle or stacking configuration may provide a route to engineer nonreciprocal superconducting transport.^3–8^ However, a general and reproducible connection between θ_twist_ and JDE remains unresolved, partly because interfacial imperfections^17–20^ can strongly modify Josephson transport even in nominally well-aligned vdW structures.

In practice, achieving uniform and atomically clean interfaces in vdW JJs remains challenging, and variations in interface transparency, contamination, or local structural disorder^17–19^ can significantly alter both the magnitude and symmetry of the supercurrent.^20^ These extrinsic effects can obscure intrinsic twist-angle-dependent behavior and complicate the interpretation of reported diode-like responses in similar systems.^3–8^

In this work, we systematically investigate NbSe_2_/NbSe_2_ vertical Josephson junctions fabricated with controlled rotational alignment and high interfacial cleanliness^21–24^ (Figure 1a). Using high-resolution transmission electron microscopy (HR-TEM) (Figure 1b), we directly determine θ_twist_ and assess interface structure at the atomic scale. We find that while the Josephson coupling strength, quantified by the average critical current, defined as $I_{\mathrm{avg}}=\frac{I_{c}^{+}+\left|I_{c}^{-}\right|}{2}$ , and *I*_avg_ *R*_n_ product, exhibits a clear dependence on θ_twist_ consistent with momentum-space matching,^6,7^ the supercurrent nonreciprocity remains negligibly small across all devices and shows no systematic twist-angle dependence. Notably, weak diode-like signals emerge only in junctions with reduced transparency^2,6,7,12^ and enhanced interfacial disorder.^20^ Deliberate degradation of the interface further amplifies this apparent nonreciprocity, despite the absence of any change in twist angle or evidence of a distinct superconducting state. Across all devices, η remains small and θ_twist_-independent, even in junctions with widely varying Josephson coupling strength. These results establish a clear decoupling between Josephson coupling and supercurrent nonreciprocity in twisted NbSe_2_/NbSe_2_ junctions. They further demonstrate that interface quality,^17–20^ rather than twist-angle-controlled momentum matching,^2,6,7,12^ dominates the observed diode response. Our findings provide a critical benchmark for interpreting previously reported Josephson diode effects in vdW superconducting systems^3–8^ and highlight the necessity of distinguishing intrinsic symmetry-breaking physics from extrinsic interface-induced artifacts.

**Figure 1:**
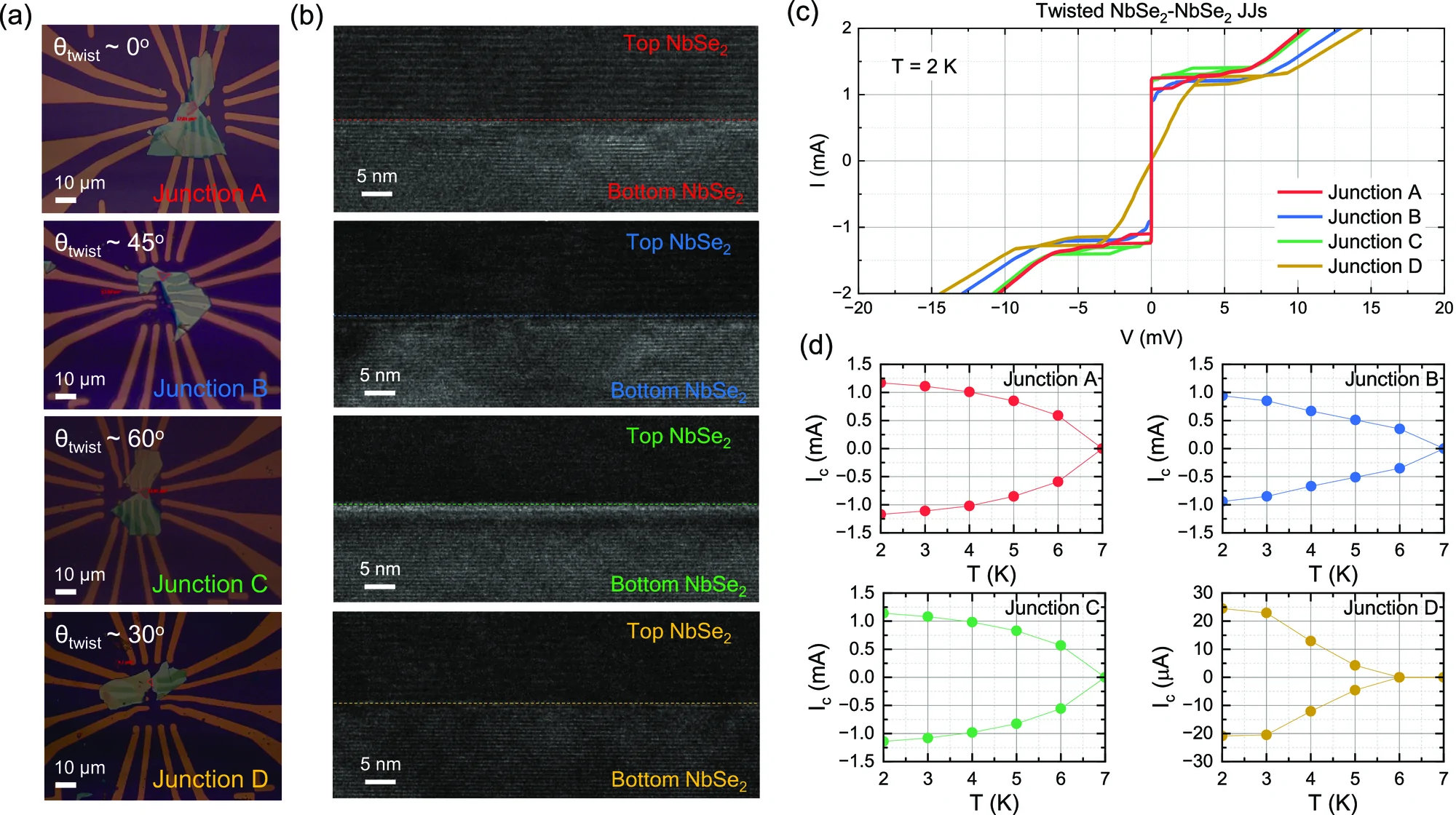
Structural characterization and electrical transport properties of NbSe_2_/NbSe_2_ van der Waals Josephson junctions. (a) Optical micrographs of junctions A–D with nominal twist angles of 0°, 45°, 60°, and 30°, respectively. (b) Cross-sectional high-resolution transmission electron microscopy (HR-TEM) images of the NbSe_2_/NbSe_2_ interfaces for junctions A–D. (c) Current–voltage *I*–*V* characteristics of junctions A–D measured at 2 K. (d) Critical current *I*_c_ as a function of temperature *T* for junctions A–D.

We first investigate the twist-angle-dependent transport properties of NbSe_2_/NbSe_2_ Josephson junctions by measuring their *I*–*V* curves at *T* = 2 K (Figure 1c). In the case of junction A with θ_twist_ ≈ 0° (Figure 1c), the critical current *I*_c_ is determined to be 1.12 mA, and junction A exhibits clear hysteresis, indicative of underdamped Josephson dynamics. To analyze the hysteretic behavior, the junction capacitance is estimated using a parallel-plate approximation *C* = ε_0_ε_r_ *A*/*d*. With a junction area *A* of 17.01 μm^2^ and an assumed interlayer spacing *d* of 0.6 nm, the effective capacitance is *C*_eff_ = 0.25 pF. Within the resistively and capacitively shunted junction (RCSJ) model,^25–29^ the McCumber parameter β = (2e*I*_c_ *R*_n_^2^ *C*)/ℏ is calculated using the extracted *C*_eff_ = 0.25 pF, the normal-state resistance *R*_n_ = 5.21 Ω obtained from the differential resistance in the quasiparticle regime, and ℏ = 1.055 × 10^–34^ J s is the reduced Planck constant. The resulting value β = 7.1 confirms that junction A operates in the underdamped regime and therefore a hysteretic *I*–*V* characteristic is expected.^25–29^ In contrast, junction D with θ_twist_ ≈ 30° exhibits a much lower β value (=0.14), indicating overdamped, nonhysteretic behavior, consistent with recent reports of overdamped vdW Josephson junctions engineered through geometrical and interfacial control.^27–29^ To further evaluate possible thermal contributions, we consider the dissipated power parameter *I*_c_^2^ *R*_n_, representing Joule heating at the switching point. We observe that hysteresis tends to decrease with reduced dissipated power, suggesting that both intrinsic Josephson dynamics (as predicted by the RCSJ model) and self-heating effects contribute to the switching behavior. This implies that the observed electrical response is also sensitive to the quality and transparency of the junction interface.^27–29^

To further understand the twist-angle-dependent transport characteristics, we compare the experimental results with the Ambegaokar–Baratoff (AB) relation,^30^ which describes BCS-type tunneling in S/I/S junctions and serves as a benchmark for evaluating phase-coherent coupling strength, $I_{c}R_{n}=\frac{\pi \Delta \left(T\right)}{2e}\tanh\left[\frac{\Delta \left(T\right)}{2k_{B}T}\right]$ , where Δ­(*T*) is the superconducting energy gap for a given *T* and *k*_B_ is the Boltzmann constant.^25–29^ At *T* = 0 K, this expression reduces to $I_{c}R_{n}=\frac{\pi \Delta \left(0\right)}{2e}$ . Using the effective average gap Δ_eff_(0) = 1.1–1.3 meV for 2H-NbSe_2_, which exhibits multiband superconductivity,^31^ we obtain a zero-temperature AB reference value of *I*_c_ *R*_n_ = 1.7–2.0 mV. Comparing the experimental results with this benchmark, we find that the junctions A–C exhibit the *I*_c_ *R*_n_ values of 4.6–6.2 mV at 2 K, exceeding the AB reference range. Such enhancement is often associated with highly transparent Josephson interfaces and transport approaching the clean short-junction or ballistic limit, where the conventional SIS tunneling description is no longer sufficient.^25–29^ In contrast, junction D shows a significantly reduced value (*I*_c_ *R*_n_ = 0.26 mV at 2 K), consistent with suppressed and weak coupling.

An analysis based on the Josephson coupling energy, $E_{J}=\frac{\hbar I_{c}}{2e}$ , which characterizes the strength of phase coupling between two superconductors in a Josephson junction, further supports this conclusion when compared with the thermal energy *k*_B_ *T*. Since junctions A–C exhibit much larger *I*_c_, they correspond to $\frac{E_{J}}{k_{B}T}∼10^{4}$ , reinforcing the argument of strong phase-coherent coupling.^32,33^ In contrast, junction D, with its much smaller *I*_c_, still satisfies $\frac{E_{J}}{k_{B}T}∼10^{2}$ , but with a significantly reduced ratio, indicating comparatively weaker phase coupling.

To elucidate the intrinsic or extrinsic origin of JDE in twisted NbSe_2_/NbSe_2_ vertical junctions, we systematically investigate the evolution of the differential resistance d*V*/d*I* as a function of *I* and μ_0_ *H*_∥_. Figure 2a presents the d*V*/d*I*(*I*, μ_0_ *H*_∥_) contour map of junction A measured over the μ_0_ *H*_∥_ range of −3 to +3 T. The blue regions correspond to the zero-resistance superconducting state. The superconducting phase boundary exhibits nearly perfect symmetry with respect to both current and magnetic-field polarity, indicating negligible nonreciprocal supercurrent transport and the absence of a measurable JDE. Qualitatively similar behavior is observed for junctions B and C (Figure 2b and c). In all three junctions, the superconducting region gradually decreases with increasing field magnitude and follows an approximately symmetric dependence that can be described by a broadened sinc-like or parabolic envelope. In contrast, junction D exhibits a substantially stronger suppression of superconductivity under μ_0_ *H*_∥_, accompanied by an irregular evolution of the superconducting phase boundary in the low-field regime (|μ_0_ *H*_∥_| < 0.3 T). This behavior differs markedly from that of junctions A–C and suggests a reduced Josephson coupling strength associated with a less transparent interface. Such an interpretation is consistent with the significantly lower *I*_c_ *R*_n_ product and *E*_J_ inferred for junction D, indicating weakened phase coherence across the junction.^25–29^ The enhanced magnetic-field sensitivity of junction D and irregular low-field response of junction D are attributed to a more weakly phase-coupled Josephson junction, likely arising from a combination of largest Fermi-surface misalignment (i.e., approaching θ_twist_ = 30°)^2,6,7,12^ and increased interfacial inhomogeneity.^17–20^ We further note that the irregular evolution of the superconducting phase boundary in this device is influenced by thermal rounding of the current–voltage characteristics near the switching transition, which becomes more pronounced in the weak-coupling regime.^25,26^

**Figure 2:**
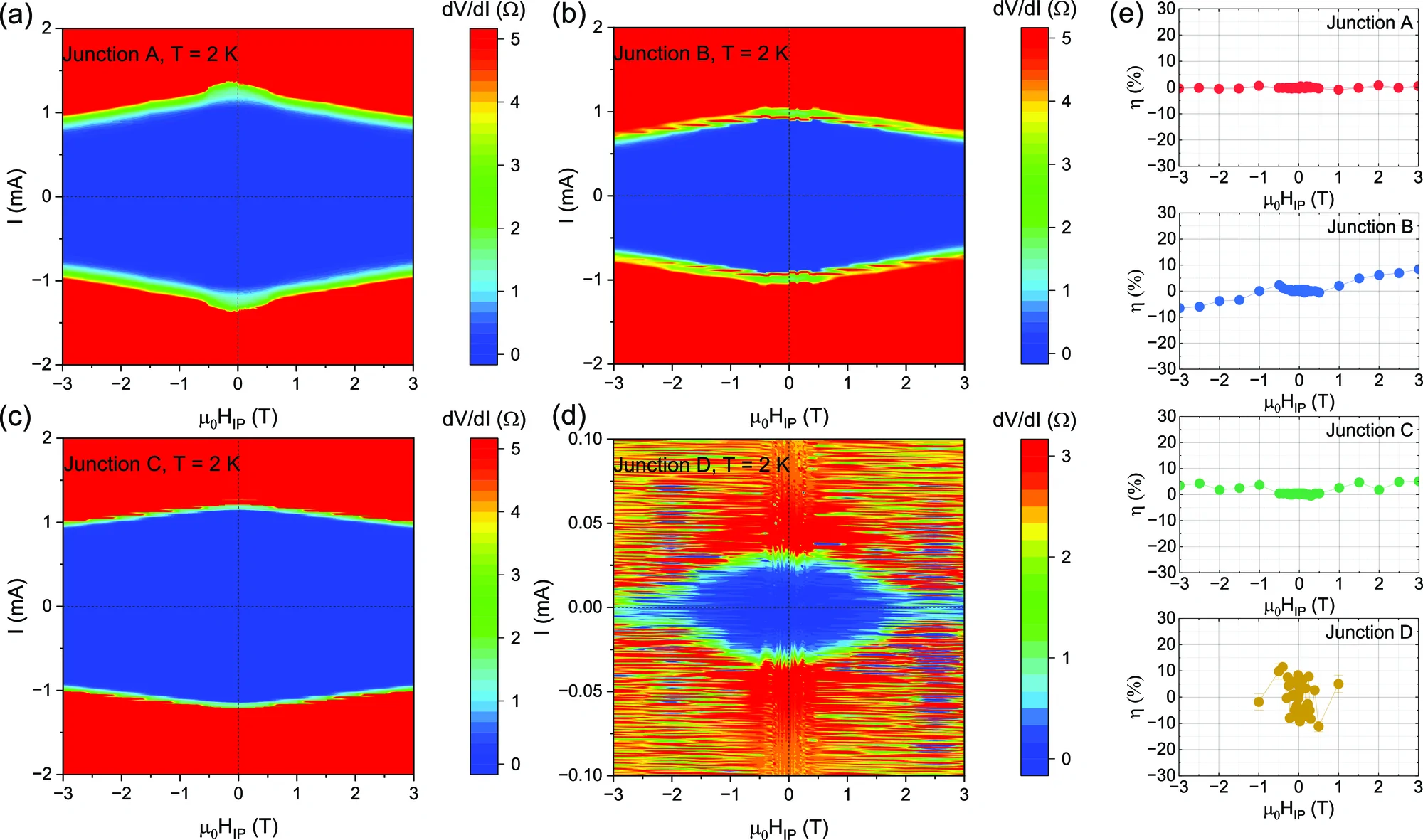
Magnetic-field dependence of superconducting transport and diode efficiency. (a–c) Differential-resistance d*V*/d*I* contour maps as functions of bias current *I* and in-plane magnetic field μ_0_ *H*_∥_ for junctions A–C measured at 2 K over the field range of −3 T ≤ μ_0_ *H*_∥_ ≤ 3 T. Blue regions correspond to the superconducting state with near-zero differential resistance. (d) Differential-resistance contour map for junction D measured under the same conditions. (e) Diode efficiency η extracted from the positive and negative critical currents (*I*_c_^+^, *I*_c_^–^) as a function of μ_0_ *H*_∥_ for junctions A–D.

The magnetic-field dependence of the diode efficiency η­(μ_0_ *H*_∥_) provides additional insight into the origin of nonreciprocal transport (Figure 2e). Junction A exhibits negligible η over the entire field range, within the experimental resolution of our measurement system, confirming the absence of measurable nonreciprocal supercurrent. For junctions B and C, η remains near zero at low magnetic fields and increases only slightly at higher fields, remaining below 8% throughout the measured range. In contrast, junction D displays a finite and highly irregular η­(μ_0_ *H*_∥_) in the low-field regime, consistent with its anomalous magnetic-field response. The correlation between the irregular magnetic-field response and the enhanced apparent diode signal (up to 11%) in junction D suggests that the observed nonreciprocity originates primarily from extrinsic interface-related effects rather than intrinsic θ_twist_-dependent superconducting physics.^6,7,12^ Taken together, these results demonstrate that all investigated junctions exhibit negligible diode efficiencies irrespective of twist angle, highlighting that local interface inhomogeneity can produce apparent nonreciprocal transport signatures that may obscure the intrinsic behavior of twisted NbSe_2_/NbSe_2_ Josephson junctions.^4,15,16^

To further examine the origin of JDE in twisted NbSe_2_/NbSe_2_ vertical junctions, we investigate the angular dependence of d*V*/d*I* map and η under a fixed in-plane magnetic field of μ_0_ *H*_∥_ = 0.07 T. This field is selected because magnetic-field-induced transport features are most clearly resolved in this regime while the junction’s superconductivity is retained for all the devices studied. Figure 3a–d shows the evolution of the d*V*/d*I* maps as the in-plane field angle γ varies in 10° increments. Junctions A–C exhibit nearly γ-independent superconducting phase boundaries, with the critical-current branches *I*_c_^±^ remaining essentially unchanged throughout the full angular range. The absence of any discernible modulation indicates that the superconducting transport in these junctions is largely insensitive to the orientation of the applied μ_0_ *H*_∥_. Such behavior is consistent with the highly symmetric field response observed in the magnetic-field-strength-dependent measurements (Figure 2) and suggests that neither θ_twist_ nor the field orientation induces measurable nonreciprocal supercurrent transport in these devices. In contrast, junction D displays a markedly different response. The d*V*/d*I* map reveals a pronounced zigzag-like modulation of the superconducting phase boundary with γ, indicating enhanced sensitivity to the magnetic-field orientation. This behavior is accompanied by substantial variations in the extracted diode efficiency, implying that the transport properties of junction D are strongly influenced by local interfacial characteristics.^17–20^ The corresponding angular dependence of the diode efficiency η­(γ) is summarized in Figure 3e. Junction A exhibits a finite but extremely small η over the entire angular range, remaining close to the experimental resolution limit. Junctions B and C show η values that are nearly zero and independent of γ, further confirming the absence of an intrinsic JDE. By contrast, junction D exhibits a significantly larger apparent diode response, reaching a maximum η of approximately 11%. Notably, however, the angular dependence does not follow a well-defined symmetry or 60° periodicity expected from an intrinsic twist-angle-related mechanism.^2,6,7,12^ Instead, η­(γ) varies irregularly with field angle and remains finite across the entire angular range. The absence of systematic angular modulation in junctions A–C, together with the irregular behavior observed in junction D, suggests that the measured nonreciprocal response is not governed by θ_twist_-dependent superconducting physics. Rather, the enhanced η observed in junction D is more consistent with extrinsic effects arising from interface inhomogeneity^17–20^ and reduced junction transparency.^2,6,7,12^

**Figure 3:**
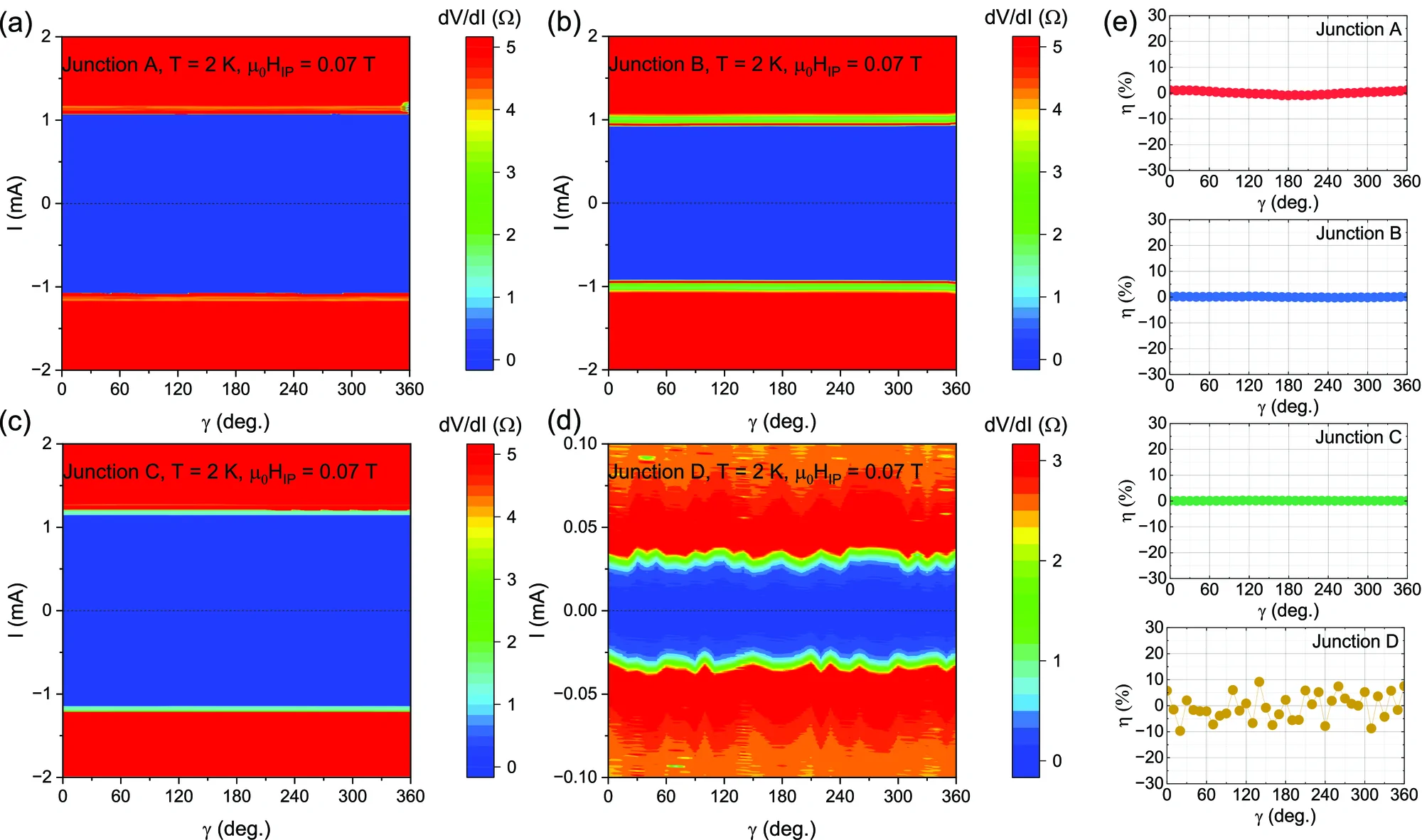
Angular dependence of superconducting transport and diode efficiency under an in-plane magnetic field. (a–c) Differential-resistance d*V*/d*I* contour maps as functions of bias current *I* and in-plane magnetic-field angle γ for junctions A–C measured at 2 K under a fixed in-plane field of μ_0_ *H*_∥_ = 0.07 T. (d) Differential-resistance contour map for junction D measured under the same conditions. (e) Diode efficiency η as a function of γ for junctions A–D, extracted from the corresponding critical currents (*I*_c_^+^, *I*_c_^–^).

To further clarify the origin of the apparent nonreciprocal supercurrent response, we investigate the temperature dependence of d*V*/d*I* and η in the NbSe_2_/NbSe_2_ vertical Josephson junctions. Figure 4a shows the d*V*/d*I* map of junction A as a function of *I* and *T*, measured under a fixed μ_0_ *H*_∥_ = 0.07 T. The dark-blue region corresponds to the zero-resistance superconducting state of the junction. As *T* increases, the superconducting phase boundary gradually shrinks and ultimately disappears above the junction’s superconducting transition temperature, reflecting the progressive suppression of Josephson coupling by thermal fluctuations.^25,26^ Junctions B–D show similar *T* dependences. In all devices, the superconducting phase boundary decreases monotonically with increasing *T* and vanishes near their respective transition temperature. Despite the markedly different magnetic-field-strength and magnetic-field-angle responses observed for junction D, its *T* evolution closely resembles that of the strongly coupled junctions. No anomalous *T*-dependent features unique to junction D are observed. This similarity indicates that all investigated devices share the same underlying superconducting transport mechanism and that the distinct behavior of junction D originates primarily from interfacial properties^17–20^ rather than from a fundamentally different superconducting state.^7^

**Figure 4:**
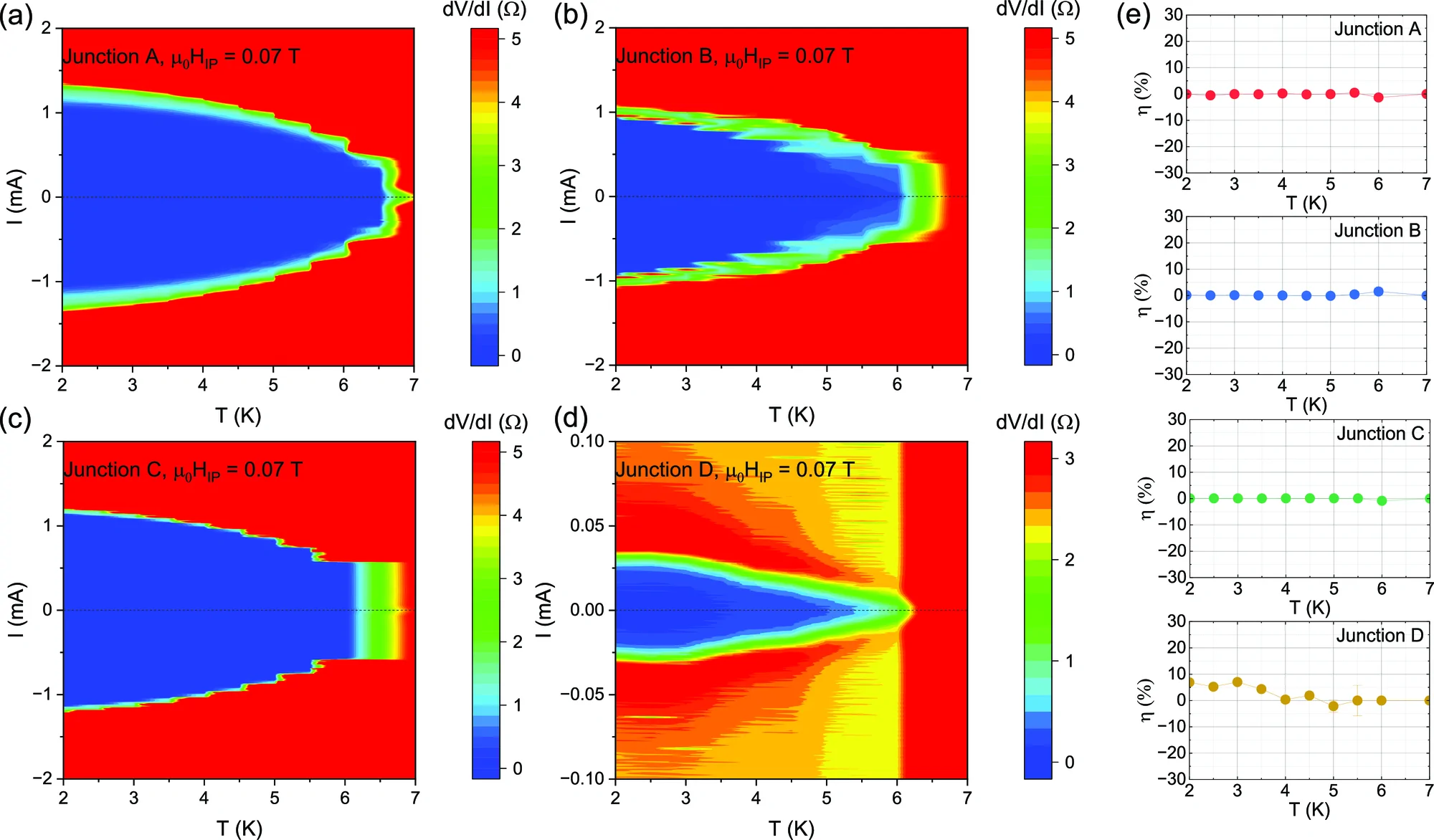
Temperature dependence of superconducting transport and diode efficiency. (a–d) Differential-resistance d*V*/d*I* contour maps as functions of bias current *I* and temperature *T* for junctions A–D measured under a fixed in-plane magnetic field of μ_0_ *H*_∥_ = 0.07 T. (e) Diode efficiency η as a function of *T* for junctions A–D, extracted from the corresponding critical currents (*I*_c_^+^, *I*_c_^–^).

The temperature dependence of the diode efficiency η­(*T*) is summarized in Figure 4e. For the strongly coupled junctions A–C, η remains close to zero throughout the entire measured *T* range, confirming the absence of measurable nonreciprocal supercurrent transport. By contrast, junction D exhibits a finite η that gradually decreases with increasing *T* and approaches zero near the transition temperature. The suppression of η­(*T*) closely follows the reduction of the superconducting switching current (Figure 1d), indicating that the apparent nonreciprocal response weakens as superconductivity is thermally suppressed. Importantly, η in junction D does not reveal any additional characteristic *T* dependence^7^ or anomalous behavior that would suggest an intrinsic diode mechanism associated with the twist angle. Therefore, although junction D exhibits a larger apparent diode response than the other devices, its overall *T* evolution remains consistent with conventional Josephson behavior.^25,26^

Figure 5a,b provides a direct comparison between the θ_twist_ dependences of Josephson coupling, quantified by the characteristic voltage *V*_c_, and supercurrent nonreciprocity, characterized by η. Here, *V*_c_ is defined as *I*_avg_ *R*_n_, while η represents the diode efficiency attained under a fixed μ_0_ *H*_∥_ = 0.07 T for each junction. As shown in Figure 5a, the Josephson coupling strength exhibits a pronounced dependence on θ_twist_. Junctions with θ_twist_ close to the crystallographically equivalent orientations (∼0° and ∼60°) show substantially larger *V*_c_ than those with intermediate θ_twist_. This behavior is consistent with the expected momentum-conserving Josephson tunneling picture, in which the overlap between the Fermi surfaces of the two NbSe_2_ electrodes depends sensitively on their relative crystallographic orientation.^2,6,7,12^ Near symmetry-equivalent stacking configurations, the overlap of electronic states is maximized, resulting in enhanced Cooper-pair transmission and stronger Josephson coupling.^2,6,7,12^ In contrast, larger momentum mismatch at intermediate twist angles suppresses coherent tunneling and reduces the critical current. Within the *c*-axis twist-junction framework,^12^ the angular dependence of the Josephson coupling can be expressed as *I*_avg_ *R*_n_(θ_twist_) ∝ ∫ d ***k*** *T*_***k***_^2^Δ­(***k***)­Δ­(*R*_θ_twist__ ***k***), where the integral is taken over the Fermi surface, *R*_θ_twist__ rotates momentum by the twist angle θ_twist_, *T*_***k***_ is the tunneling matrix element, Δ­(***k***) is the superconducting gap. For 2H-NbSe_2_, Δ­(***k***) inherits a 6-fold symmetry from the underlying hexagonal lattice.^13,14^ As a result, *I*_avg_ *R*_n_(θ_twist_) exhibits periodic modulations governed by crystal symmetry and momentum-selective tunneling, approximately captured by a 60°-periodic form such as *I*_avg_ *R*_n_(θ_twist_) ∝ |cos­(3θ_twist_)| (dashed line in Figure 5a). The observed trend therefore confirms that the Josephson coupling strength in clean NbSe_2_/NbSe_2_ vertical junctions remains strongly governed by twist-angle-controlled momentum-space alignment.^2,6,7,12^

**Figure 5:**
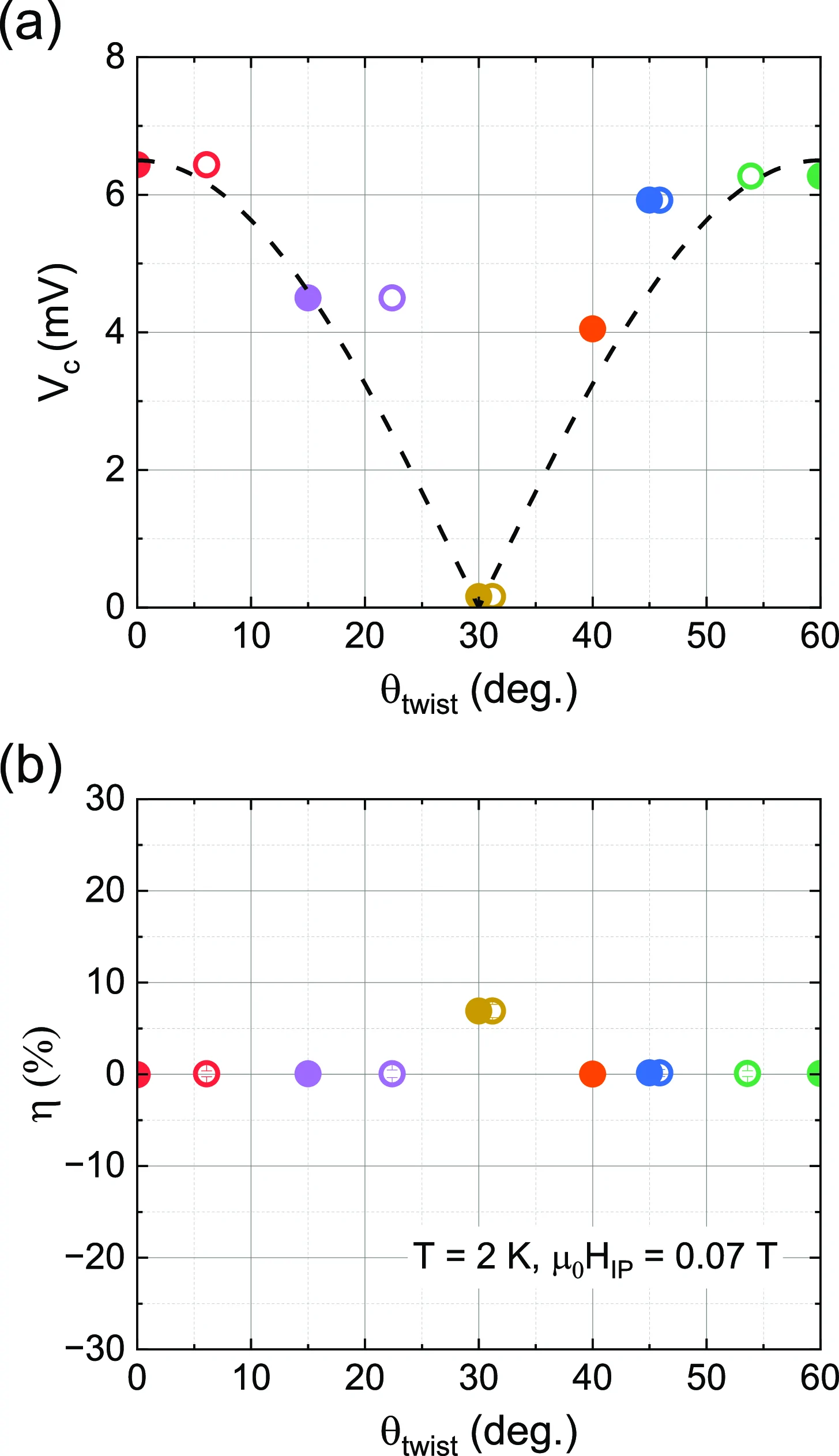
Twist-angle dependence of Josephson coupling and supercurrent nonreciprocity. (a) Characteristic voltage *V*_c_ (=*I*_avg_ *R*_n_) as a function of twist angle θ_twist_ for junctions A–F. Note that detailed data for junctions E and F are presented in the Supporting Information. The dashed line in panel a exhibits a periodic modulation of *I*_avg_ *R*_n_(θ_twist_) ∝ |cos­(3θ_twist_)|, reflecting the crystal symmetry and momentum-selective tunneling, and is approximately captured by a 60°-periodic form. (b) Diode efficiency η as a function of θ_twist_ for junctions A–F, measured under a fixed in-plane magnetic field of μ_0_ *H*_∥_ = 0.07 T at 2 K. The data reveal a pronounced twist-angle dependence of the Josephson coupling, whereas the supercurrent nonreciprocity remains small and exhibits no systematic dependence on θ_twist_. In both panels a and b, closed (open) symbols indicate nominal (estimated) twist angles; further details are provided in the Supporting Information.

In striking contrast, the Josephson supercurrent nonreciprocity exhibits no systematic dependence on θ_twist_ (Figure 5b). For all investigated junctions, η remains small and does not follow the trend observed for *V*_c_. Although junction D exhibits a somewhat larger apparent diode efficiency than the other devices, this enhancement occurs despite its significantly weaker Josephson coupling and does not correlate with any specific θ_twist_. Furthermore, as demonstrated above, the enhanced η of junction D is accompanied by irregular magnetic-field and angular responses, suggesting that it originates from extrinsic interface-related effects^17–20^ rather than an intrinsic θ_twist_-induced mechanism.^2,6,7,12^ Consequently, the overall distribution of η across the measured θ_twist_ range indicates that the nonreciprocal supercurrent response is essentially independent of θ_twist_.

The contrasting behaviors of *V*_c_ and η provide an important insight into the physics of twisted NbSe_2_/NbSe_2_ vertical Josephson junctions. While twist-angle engineering effectively modulates the Josephson coupling strength through momentum-space matching of the superconducting electrodes, it does not generate a corresponding enhancement of nonreciprocal supercurrent transport when the interfaces are chemically clean and structurally uniform. Instead, any residual diode-like response appears to be dominated by local interface inhomogeneity, transparency variations, or other extrinsic factors.^17–20^ These findings establish a clear distinction between the mechanisms governing Josephson coupling and supercurrent nonreciprocity and indicate that large Josephson diode effects reported in similar vdW junction systems^3–8^ should be carefully evaluated in terms of interface quality^17–20^ in addition to twist-angle considerations.^2,6,7,12^

To further clarify the origin of the apparent nonreciprocal response, we focus on the weak-coupling regime, where Josephson transport is strongly suppressed and becomes highly sensitive to local interface conditions. In particular, junctions with twist angles near 30° exhibit the smallest critical currents and the lowest *I*_c_ *R*_n_ products, consistent with reduced momentum-space overlap and weakened phase coherence between the NbSe_2_ electrodes.^2,6,7,12^ In this regime, we find that the measured diode efficiency can be substantially enhanced when the junction interface is intentionally degraded. Specifically, when the post-stacking bakeout step is omitted,^4,9–11^ the junctions exhibit a more pronounced apparent nonreciprocal response within a restricted magnetic-field window (Figure 6a–c). In these devices, the extracted diode efficiency η can reach values as high as ∼30%. Importantly, this enhancement does not coincide with any systematic change in θ_twist_ or with signatures of a distinct superconducting state.

**Figure 6:**
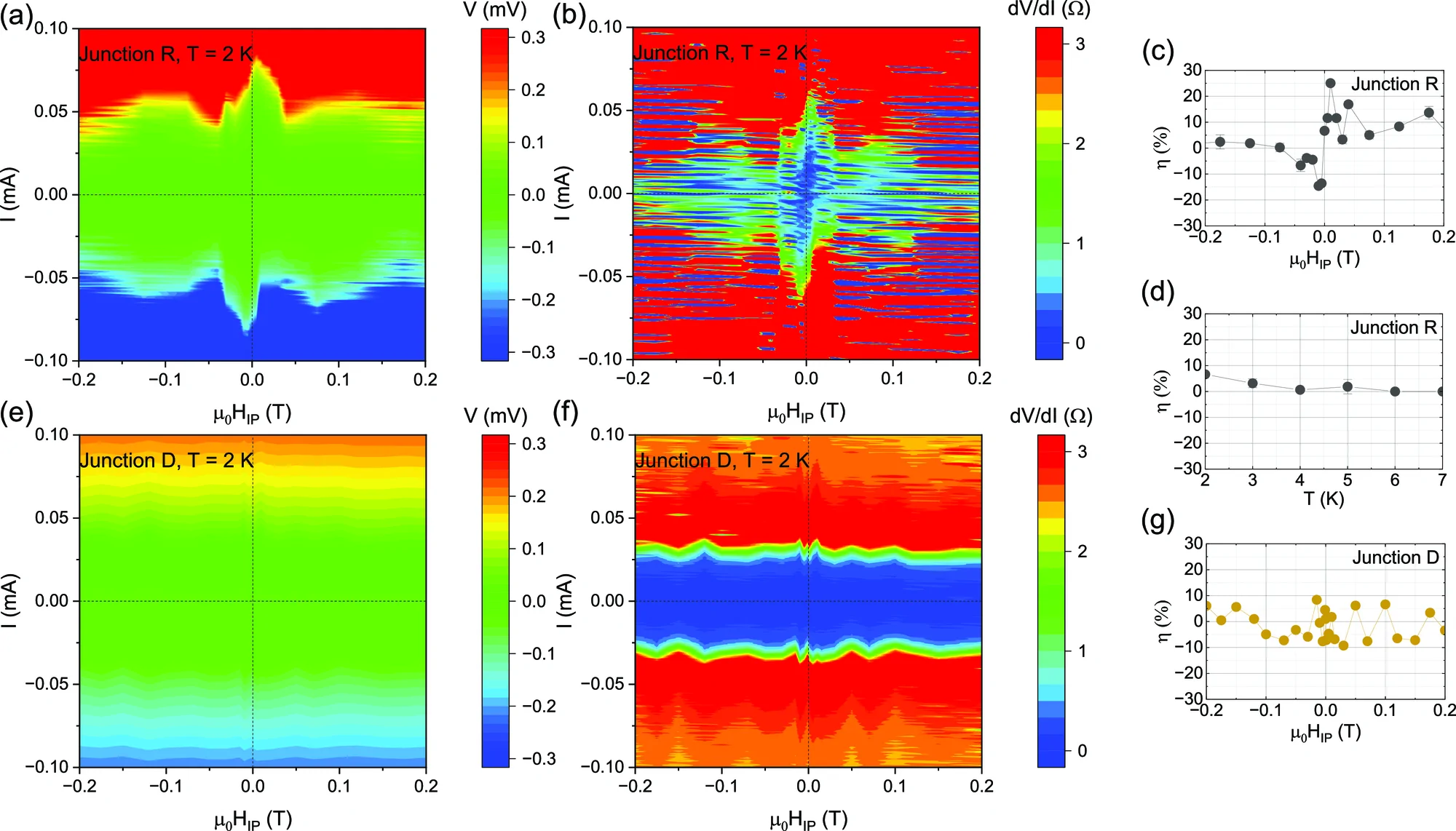
Interface-degradation-induced apparent Josephson diode effect. (a and b) Voltage *V* and differential-resistance d*V*/d*I* contour maps as functions of bias current *I* and in-plane magnetic field μ_0_ *H*_∥_ for junction R, measured at 2 K over the field range of −0.2 T ≤ μ_0_ *H*_∥_ ≤ 0.2 T. The blue region in panel b corresponds to the superconducting state with near-zero differential resistance. (c) Diode efficiency η extracted from the positive and negative critical currents (*I*_c_^+^, *I*_c_^–^) as a function of μ_0_ *H*_∥_ for junction R. (d) Diode efficiency η as a function of *T* for junction R. (e–g) Corresponding measurements for junction D under the same conditions, shown for direct comparison. Note that the degraded-interface junction R exhibits a substantially enhanced apparent diode response and a more irregular magnetic-field dependence than junction D, highlighting the strong influence of interface quality on the observed nonreciprocal transport.

Instead, the large apparent η is accompanied by strongly irregular and nonmonotonic magnetic-field dependence of the critical current, as well as enhanced sensitivity to small variations in field strength. Such behavior indicates a breakdown of uniform Josephson coupling across the junction area, consistent with spatially inhomogeneous interface transparency^17–20^ and reduced phase stiffness.^25,26^ In this situation, current distribution becomes highly nonuniform, and local switching events can be influenced by microscopic disorder^20,34^ and localized heating near the critical current. These effects can produce different effective switching thresholds for positive and negative bias currents, thereby mimicking a diode-like response.

Consistent with this picture, weakly coupled junctions generally exhibit irregular magnetic-field dependence of the rectification parameter without a well-defined even or odd parity. This behavior may arise from spatially inhomogeneous interface transparency, asymmetric current distributions, trapped flux, or other sources of junction asymmetry that introduce multiple current paths with different effective areas and local phase responses. However, identifying the specific microscopic mechanism responsible for the apparent diode response will require further systematic investigation.

The observation that the strongest apparent nonreciprocity arises in the weakest and most disordered junctions provides straightforward evidence that the enhanced Josephson diode effect in these devices is not intrinsic in origin. Rather, it emerges from extrinsic factors associated with interface inhomogeneity,^17–20,34^ including spatial variations in transparency,^2,6,7,12^ local suppression of superconductivity, and nonuniform current flow. This interpretation is fully consistent with the θ_twist_-independent behavior of η discussed above, and it reinforces the conclusion that crystallographic misalignment does not play a primary role in generating nonreciprocal supercurrent transport in clean NbSe_2_/NbSe_2_ vertical Josephson junctions. Taken together, these results demonstrate that apparent diode effects can be artificially amplified in the weak-coupling regime due to interface disorder,^17–20^ rather than arising from an intrinsic symmetry-breaking mechanism^2,6,7,12^ linked to θ_twist_.

Different superconductors and junction geometries may involve additional symmetry-breaking mechanisms, including spin–orbit coupling, magnetic order, strain, or other sources of intrinsic nonreciprocity. The significance of our result is therefore that within a controlled van der Waals Josephson platform, the effects of twist angle on Josephson coupling can be experimentally distinguished from those of extrinsic interface disorder^20,34^ on nonreciprocal transport.

In summary, we have systematically investigated the role of θ_twist_ in determining both the Josephson coupling strength and the nonreciprocal supercurrent response in NbSe_2_/NbSe_2_ vertical JJs fabricated via a dry transfer technique with improved interface cleanliness. While the critical current and Josephson coupling energy exhibit a clear dependence on θ_twist_, consistent with variations in momentum-space overlap between the superconducting layers,^2,6,7,12^ the nonreciprocal supercurrent response remains negligibly small across all measured devices and shows no systematic dependence on θ_twist_. Despite observing a wide range of junction transparencies, from strongly coupled junctions with anomalously high *I*_c_ *R*_n_ values exceeding the AB limit^30^ to weakly coupled junctions approaching the conventional tunneling regime, the measured η remains near zero in all cases. Only a weak and nonuniversal enhancement of asymmetry is observed in weakly coupled junctions within a limited magnetic-field window, suggesting that any apparent diode-like behavior is not intrinsic to θ_twist_ but is instead highly sensitive to interfacial inhomogeneity and junction quality.^17–20^ These results demonstrate that twist-angle engineering^2,6,7,12^ alone is not sufficient to induce robust nonreciprocal superconducting transport in NbSe_2_-based vertical Josephson junctions when high-quality, chemically clean interfaces are realized. Instead, our findings highlight interface transparency^2,6,7,12^ and microscopic disorder^17–20,34^ as the dominant factors governing any residual diode response. This provides a revised framework for interpreting previously reported large JDEs in similar van der Waals systems^6–8^ and establishes an important benchmark for future studies aiming to exploit layered superconductors for controlled nonreciprocal superconducting electronics.

## Supplementary Material



## Data Availability

All data required to evaluate the conclusions of this study are included in the article and/or the Supporting Information.

## Notes

Sections S1–S6 include the following: fabrication process and transport measurements of the van der Waals Josephson junctions, optical microscopy and atomic force microscopy characterization of the fabricated devices, high-resolution transmission electron microscopy characterization used to determine the relative crystal orientation and twist angle, Josephson properties of junctions E and F with nominal twist angles of 15° and 40°, respectively, Josephson properties of junction G with a nominal twist angle of 30°, and comparison with a recent theory (PDF)
