# Supplementary material for: Decoupling Josephson Coupling and Supercurrent Nonreciprocity in Twisted NbSe2/NbSe2 van der Waals Junctions
Source: Nano Lett. 2026 Oct 23;26(43):14993–5001. doi: 10.1021/acs.nanolett.6c03685 (PMC13741916; doi:10.1021/acs.nanolett.6c03685)
Supplement: Supplementary file 1 [file nl6c03685_si_001.pdf]

Supplementary Information

Decoupling Josephson Coupling and Supercurrent Nonreciprocity in Twisted $NbSe_2/NbSe_2$ van der Waals Junctions

Seung-Gi Lee[1], Kwon-Neong Jung[1], Jae-Keun Kim[3], Jae-Chun Jeon[2], Jiho Yoon[2], Stuart S. P. Parkin[2*], and Kun-Rok Jeon[1*]

[1]*Department of Physics, Chung-Ang University (CAU), 06974 Seoul, Republic of Korea*

[2]*Max Planck Institute of Microstructure Physics, Weinberg 2, 06120 Halle (Saale), Germany*

[3]*Department of Physics, University of Seoul, 02504 Seoul, Republic of Korea*

*To whom correspondence should be addressed: stuart.parkin@halle-mpi.mpg.de, jeonkunrok@gmail.com

This PDF file includes:

Supplementary Text

Sections S1–S6: fabrication process and transport measurements of the van der Waals Josephson junctions; optical microscopy and atomic force microscopy characterization of the fabricated devices; high-resolution transmission electron microscopy characterization used to determine the relative crystal orientation and twist angle; Josephson properties of Junctions E and F with nominal twist angles of 15° and 40°, respectively; Josephson properties of Junction G with a nominal twist angle of 30°; and comparison with a recent theory.

Figs. S1 to S6

References (*S1-S7*)

Section S1: Fabrication process and transport measurements of the van der Waals Josephson junctions

The $NbSe_2/NbSe_2$ van der Waals (vdW) vertical Josephson junctions were fabricated using a dry-transfer technique in a controlled inert environment[S1-S4] (Figure S1). Bulk 2H-$NbSe_2$ single crystals were purchased from HQ Graphene and mechanically exfoliated onto a SiO_2(300 nm)/Si substrate using polydimethylsiloxane (PDMS) stamps. Individual flakes required for the heterostructure were identified by optical microscopy. Flakes exhibiting uniform thickness, smooth surfaces, and well-defined straight edges were selected for device fabrication. The selected flakes were sequentially picked up using a polycarbonate (PC)-coated, dome-shaped PDMS stamp to assemble the $NbSe_2/NbSe_2$ stack. Prior to flake assembly, Ti(5 nm)/Au(50 nm) electrodes were patterned on a separate SiO_2(300 nm)/Si substrate using conventional photolithography, ion-beam sputtering, and lift-off processes. The assembled $NbSe_2/NbSe_2$ stack was then transferred onto the pre-patterned electrodes using a dry-transfer system (HQ Graphene) equipped with a rotational stage. During stacking, the long, straight edges of the flakes served as references for approximate crystallographic alignment. The relative crystal orientation between the top and bottom flakes was controlled by rotating the transfer stage before bringing the assembled stack into contact. To minimize oxidation and contamination, all transfer procedures were performed inside a nitrogen-filled glove box with H_2O and O_2 concentrations maintained below 0.5 ppm. Following assembly, the devices were annealed under vacuum at a moderate temperature to improve interfacial contact and remove residual adsorbates introduced during fabrication.

The current-voltage I–V curves of the fabricated $NbSe_2/NbSe_2$ vertical JJs are measured in a Quantum Design Physical Property Measurement System over a temperature T range from 300 K down to 2 K. Four-probe I–V measurements are performed using a Keithley 6221 current source and a Keithley 2182 A nanovoltmeter. All working junctions exhibit Ohmic I–V

characteristics in the normal state, with resistance values in the range of 40–60 Ω at 300 K. A superconducting transition of such junctions is observed at approximately 6 K. The critical current I_c is defined either as the current at which $V(I) \approx 1$ μV or by fitting the measured *I–V* curves with $V(I) = \frac{I}{|I|}\sqrt{I^2 - I_c^2}$, following conventional Josephson-junction transport analysis[S5].

We note that θ denotes the angle between the applied $\mu_0 H_{||}$ and one edge of the Si wafer, which is intentionally cut into a rectangular shape to provide a well-defined reference direction. The exfoliated layer stack is aligned with this edge. Field-dependent diode signals are measured by applying $\mu_0 H_{||}$ along a specified θ direction. Angle-dependent diode signals are obtained by rotating the sample with respect to a fixed superconducting electromagnet while maintaining a constant $\mu_0 H_{||}$.

We emphasize that the conclusions of the present study are based on a systematic comparison of the twist-angle dependence of Josephson coupling and diode efficiency across multiple junctions prepared using the same stacking protocol under comparable glovebox conditions, rather than on the assumption of perfectly homogeneous interfaces.

Section S2: Optical microscopy and atomic force microscopy characterization of the fabricated devices

Optical microscopy was used to identify suitable $NbSe_2$ flakes and monitor each step of the device fabrication process. Representative optical images of the fabricated van der Waals (vdW) Josephson junctions are presented in Figure S2. The overlap region between the top and bottom $NbSe_2$ flakes defines the active Josephson junction area (outlined by the red solid line). Atomic force microscopy (AFM) measurements were performed in tapping mode under ambient conditions to determine the thicknesses of the constituent $NbSe_2$ layers and assess their surface

morphology. Representative AFM images and corresponding height profiles are also shown in Figure S2. The measured flake thicknesses ranged from approximately 20 to 55 nm. AFM characterization revealed atomically flat terraces with low surface roughness ($\leq$ 0.7 nm), indicating high surface quality. No noticeable surface degradation or particulate contamination was observed within the overlap regions forming the Josephson junctions. The geometrical junction area used in the transport analysis was determined from the optical images and corroborated by the corresponding AFM images.

Section S3: High-resolution transmission electron microscopy characterization of the relative crystal orientation and twist angle

High-resolution transmission electron microscopy[S6] (HR-TEM) was employed to determine the relative crystal orientation between the stacked $NbSe_2$ layers. Cross-sectional TEM specimens were prepared using a focused ion beam (FIB) lift-out method. A protective metal layer was deposited before milling to minimize ion-beam-induced damage. The specimens were thinned to electron transparency and examined using an aberration-corrected transmission electron microscope operated at 200 kV. Figure S3a shows a representative cross-sectional HR-TEM image of a fabricated $NbSe_2/NbSe_2$ junction (Junction A). The layered crystal structure and atomically sharp interface between the two $NbSe_2$ flakes are clearly resolved, as further confirmed by energy-dispersive X-ray spectroscopy (EDS) analysis (Figure S3b). No intentionally introduced tunnel barrier is observed at the interface. Instead, only a thin residual interfacial layer, attributed to unavoidable transfer-related adsorbates, is occasionally present.

The twist angle θ_{twist} was determined using a combination of fast Fourier transform (FFT) analysis of lattice-resolved cross-sectional HR-TEM images and double-tilt TEM alignment. The top and bottom $NbSe_2$ layers were independently aligned to the same in-plane crystallographic zone axis using FFT-guided double-tilt TEM alignment. Representative FFT

patterns for the top and bottom layers are shown in Figures S3c and S3d, respectively. The corresponding stage tilt coordinates (TX and TY) were recorded for each layer. The interlayer misorientation, corresponding to θ_{twist}, was calculated from the relative rotation matrix constructed from the recorded TX and TY stage tilt coordinates. Given the nearly parallel *c*-axes of the two layers, the resulting misorientation angle was taken as the interlayer θ_{twist}.

Specifically, for each layer, the orientation matrix was constructed as $\mathbf{R} = \boldsymbol{R}_y(\beta)\,\boldsymbol{R}_x(\alpha)$, where α = TX and β = TY. The stage tilt angles were converted from degrees to radians prior to the calculation. The rotation matrices about the *x*- and *y*-axes are given by $\boldsymbol{R}_x(\alpha) = \begin{bmatrix} 1 & 0 & 0 \\ 0 & \cos(\alpha) & -\sin(\alpha) \\ 0 & \sin(\alpha) & \cos(\alpha) \end{bmatrix}$ and $\boldsymbol{R}_y(\beta) = \begin{bmatrix} \cos(\beta) & 0 & \sin(\beta) \\ 0 & 1 & 0 \\ -\sin(\beta) & 0 & \cos(\beta) \end{bmatrix}$. The relative orientation between the two $NbSe_2$ layers was obtained from $\boldsymbol{R}_{\mathbf{rel}} = \boldsymbol{R}_{\mathbf{bottom}}\boldsymbol{R}_{top}^{T}$, where $\boldsymbol{R}_{\mathbf{bottom}}$ and $\boldsymbol{R}_{\mathbf{top}}$ are the orientation matrices of the top and bottom layers, respectively. The interlayer misorientation angle was then calculated from the trace of the relative rotation matrix according to $\theta_{twist} = \cos^{-1}\left[\frac{\mathrm{Tr}(\boldsymbol{R}_{\mathbf{rel}}) - 1}{2}\right]$. Note that owing to the six-fold rotational symmetry of $NbSe_2$, θ_{twist} and $60 - \theta_{twist}$ produce crystallographically equivalent lattice orientations and therefore cannot be distinguished from the TEM alignment alone. The final twist angle was consequently assigned by comparison with the nominal twist angle of the fabricated heterostructure.

The estimated values of θ_{twist} are consistent with the nominal values defined during the dry-transfer assembly process, within an experimental deviation of up to 7° (Figure 5). The uncertainty in the extracted twist angle is estimated to be approximately ±2°, primarily arising from the finite angular resolution of the FFT analysis and local variations in crystal orientation likely caused by the FIB lift-off process. These HR-TEM results confirm the high structural quality of the $NbSe_2$/$NbSe_2$ interfaces and verify that the fabricated junctions possess well-defined relative crystallographic orientations.

We note that localized defects or inhomogeneity outside the characterized regions may remain undetected. A complete spatially resolved map of disorder across the entire buried interface would require more extensive characterization, which is beyond the scope of the present work. Therefore, while the HR-TEM measurements provide direct structural information for the representative regions examined, they do not exclude the possibility of localized interfacial inhomogeneity elsewhere in the junctions.

Section S4: Josephson properties of Junctions E and F with nominal twist angles of 15° and 40°

To further verify device reproducibility and characterize junction behavior, we examine Junctions E and F, which were fabricated using the same method as the devices discussed in the main text. The nominal twist angles of ~15° and ~40°, respectively, for Junctions E and F, were estimated from the relative orientation of the long, straight edges of the top and bottom flakes. Figure S4b shows a cross-sectional HR-TEM image of Junction E. Clear lattice fringes are observed in both the top and bottom $NbSe_2$ layers, separated by a well-defined van der Waals gap at the interface. The absence of noticeable lattice distortion indicates the formation of a high-quality interface between the two flakes.

Consistent with the junctions discussed in the main text, the critical current I_c decreases with increasing temperature and vanishes near the superconducting transition temperature, as shown in Figure S4c. Figures S4d and S4e show dV/dI maps as a function of magnetic field $\mu_0 H_\parallel$ and temperature T, respectively. The superconducting phase boundary is approximately symmetric with respect to the polarity of $\mu_0 H_\parallel$ and narrows as T increases. The extracted η remains close to zero over the full range of $\mu_0 H_\parallel$ and T investigated. In contrast to Junctions D and R, where enhanced η was observed, Junction E shows no significant non-reciprocal response. These results consistently support that the enhanced η observed in those devices is

more likely associated with interfacial inhomogeneity and weakened Josephson coupling rather than the twist angle itself.

A qualitatively similar absence of the Josephson diode effect is observed in Junction F (Figures S4f–l), further supporting the conclusion that the twist angle, although it strongly influences the Josephson coupling strength, is not directly linked to supercurrent nonreciprocity in twisted $NbSe_2/NbSe_2$ van der Waals junctions.

Section S5: Josephson properties of Junction G with a nominal twist angle of 30°

To further verify device reproducibility and characterize junction behavior, we examine Junction G, which was fabricated using the same method as Junction R discussed in the main text. The nominal twist angle of ~30° was estimated from the relative orientation of the long, straight edges of the top and bottom flakes.

Similar to the weakly coupled Junction D and the deliberately degraded control Junction R, Junction G exhibits enhanced nonreciprocity, indicating that reduced interface quality is associated with a stronger diode effect. Although the detailed magnitude and magnetic-field dependence of the diode response can vary from device to device, the main qualitative features of the diode effect, including its magnetic-field and temperature dependence, show consistent behavior across weakly coupled junctions. These observations further support the close association between reduced interface quality and enhanced superconducting diode behavior.

We emphasize that the apparent onset of the diode response at approximately 4 K in Junction D should not be interpreted as a universal transition temperature for the Josephson diode effect. Similar behavior is observed in Junctions R and G, suggesting that this temperature scale is associated with the emergence of measurable Josephson transport in the

weak-coupling regime. As the temperature decreases, the Josephson current increases, making a relatively small asymmetry arising from interfacial inhomogeneity experimentally resolvable. Consequently, the onset temperature can depend on the interfacial Josephson coupling strength, junction quality, and measurement sensitivity. The ~4 K temperature scale should therefore be regarded as a device-dependent characteristic temperature rather than a universal energy scale of the diode effect.

Section S6: Comparison with a recent theory

The role of junction disorder in superconducting diode effects in the absence of Rashba coupling has recently been discussed[S7]. To examine whether the apparent diode effect observed in our experiments (Figure S6a-c) is related to the theoretical predictions presented in that work, we plotted the rectification efficiency as a function of I_{avg} for Junctions R, D, and G (Figure S6d-f). In particular, the theory predicts an enhancement of the rectification amplitude near magnetic-field values where the supercurrent amplitude is strongly suppressed[S7].

For our deliberately degraded junctions, which show enhanced non-reciprocity in certain magnetic-field regions (Figure S6a,c), we do not observe a sufficiently systematic correspondence between minima in the critical current and maxima in the rectification parameter to establish the specific correlation predicted by the theoretical model[S7]. The irregular I_{avg} dependence of the rectification efficiency (Figure S6d-f) instead suggests that spatial inhomogeneity and junction-specific current distributions may play important roles in the observed diode-like response.

Thus, while our observations are qualitatively consistent with the general picture that junction inhomogeneity can contribute to non-reciprocal transport, the present data do not provide clear quantitative evidence for the specific correlation predicted by the theoretical model[S7].

REFERENCES

[S1] Cheliotis, I.; Zergioti, I. A Review on Transfer Methods of Two-Dimensional Materials. *2D Mater.* **2024**, 11, 022004.

[S2] Frisenda, R.; Navarro-Moratalla, E.; Gant, P.; Pérez De Lara, D.; Jarillo-Herrero, P.; Gorbachev, R. V.; Castellanos-Gomez, A. Recent Progress in the Assembly of Nanodevices and van der Waals Heterostructures by Deterministic Placement of 2D Materials. *Chem. Soc. Rev.* **2018**, 47, 53–68.

[S3] Gasparutti, I.; Song, S. H.; Neumann, M.; Wei, X.; Watanabe, K.; Taniguchi, T.; Lee, Y. H. How Clean Is Clean? Recipes for van der Waals Heterostructure Cleanliness Assessment. *ACS Appl. Mater. Interfaces* **2020**, 12, 37062–37069.

[S4] Andrei, E. Y.; MacDonald, A. H. Graphene Bilayers with a Twist. *Nat. Mater.* **2020**, 19, 1265–1275.

[S5] Barone, A.; Paternò, G. Physics and Applications of the Josephson Effect; Wiley: New York, 1982.

[S6] Haigh, S. J.; Gholinia, A.; Jalil, R.; Romani, S.; Britnell, L.; Elias, D. C.; Novoselov, K. S.; Ponomarenko, L. A.; Geim, A. K.; Gorbachev, R. Cross-Sectional Imaging of Individual Layers and Buried Interfaces of Graphene-Based Heterostructures and Superlattices. *Nat. Mater.* **2012**, 11, 764–767.

[S7] Chirolli, L.; Greco, A.; Crippa, A.; Strambini, E.; Cuoco, M.; Amico, L.; Giazotto, F. Diode Effect in the Fraunhofer Pattern of Disordered Planar Josephson Junctions. *Commun. Phys.* **2025**, 8, 483.

FIGURE CAPTIONS

Figure S1. Fabrication process of the van der Waals Josephson junctions. (a) Schematic

of $NbSe_2$ flake exfoliation and selection on SiO_2 (300 nm)/Si substrates. (b) Schematic of the dry-transfer process using a PC-coated PDMS stamp to assemble the $NbSe_2/NbSe_2$ heterostructure on pre-patterned Ti/Au electrodes. (c) Schematic of the completed vertical Josephson junction and the four-terminal measurement configuration.

Figure S2. Optical microscopy and atomic force microscopy characterization of the fabricated devices. (a) Optical microscopy image of a representative fabricated $NbSe_2/NbSe_2$ vdW Josephson junction, along with the corresponding four-terminal measurement configuration. The overlap region between the top and bottom $NbSe_2$ flakes defines the active junction area. (b) AFM images of representative junction devices (A–D). AFM measurements were used to determine the thicknesses of the constituent $NbSe_2$ flakes and evaluate their surface morphology. The corresponding height profiles, extracted along the lines indicated in the AFM images, were used to determine the thicknesses of the individual $NbSe_2$ flakes. Note that for Junction A, AFM characterization was not performed prior to focused ion beam (FIB) processing because of an experimental oversight. As a result, the thicknesses of the top and bottom $NbSe_2$ flakes could not be determined by AFM after the transport measurements. Instead, their thicknesses were estimated from the high-resolution transmission electron microscopy (HR-TEM) analysis presented in Section S3.

Figure S3. High-resolution transmission electron microscopy characterization of the $NbSe_2/NbSe_2$ junction. (a) Cross-sectional HR-TEM image of a representative $NbSe_2/NbSe_2$ junction (Junction A), showing the layered crystal structure and an atomically sharp interface between the stacked $NbSe_2$ flakes. (b) Energy-dispersive X-ray spectroscopy (EDS) analysis of the $NbSe_2/NbSe_2$ junction, confirming the elemental distribution across the interface. (c) Fast Fourier transform (FFT) pattern of the top $NbSe_2$ layer extracted from the lattice-resolved

HR-TEM image. (d) FFT pattern of the bottom $NbSe_2$ layer. The twist angle θ_{twist} was determined from the relative angular difference between the crystallographic zone axes of the two $NbSe_2$ layers. (e–h), (i–l), (m–p), and (q–t) show the corresponding HR-TEM image, EDS analysis, and FFT patterns for Junctions B, C, D, and E, respectively. For each junction, the twist angle was estimated by combining FFT analysis of lattice-resolved cross-sectional HR-TEM images with double-tilt TEM alignment.

Figure S4. Josephson properties of Junctions E and F with nominal twist angles of 15° and 40°. (a) Optical micrograph of Junction E with a nominal twist angle of approximately 15°, along with the corresponding four-terminal measurement configuration. (b) Cross-sectional HR-TEM image of $NbSe_2$/$NbSe_2$ interface for Junction E. (c) Critical current I_c as a function of temperature T for Junction E. (d) Differential-resistance dV/dI contour map as a function of I and in-plane magnetic field $\mu_0H_\parallel$ for Junction E, measured at 2 K over the field range −0.2 T ≤ $\mu_0H_\parallel$ ≤ 0.2 T. (e) Differential-resistance dV/dI contour map as a function of I and T, measured under a fixed $\mu_0H_\parallel = 0.07$ T. Extracted diode efficiency η of Junction E from the corresponding critical currents (I_c^+, I_c^-), plotted as a function of $\mu_0H_\parallel$ (f) and T (g). (h–n) Corresponding measurements for Junction F with a nominal twist angle of approximately 40°: (h) optical micrograph, (i) AFM image, (j) $I_c(T)$ plot, (k) dV/dI contour map as a function of I and $\mu_0H_\parallel$, (l) dV/dI contour map as a function of I and T, and η as a function of $\mu_0H_\parallel$ (m) and T (n) for Junction F.

Figure S5. Josephson properties of Junction G with a nominal twist angle of 30°. (a) Optical micrograph of Junction G with a nominal twist angle of approximately 30°, along with the corresponding four-terminal measurement configuration. (b) AFM image of $NbSe_2$/$NbSe_2$ stacking region in Junction G. (c) Critical current I_c as a function of temperature T for Junction

G. (d) Voltage V contour map as a function of I and in-plane magnetic field $\mu_0 H_\parallel$ for Junction G, measured at 2 K over the field range −0.2 T ≤ $\mu_0 H_\parallel$ ≤ 0.2 T. (e) Voltage V contour map as a function of I and T, measured under a fixed $\mu_0 H_\parallel$ = 0.07 T. Extracted diode efficiency η of Junction G from the corresponding critical currents (I_c^+, I_c^-), plotted as a function of $\mu_0 H_\parallel$ (f) and T (g).

Figure S6. Comparison with a recent theory. (a) Differential-resistance dV/dI contour map as functions of bias current I and in-plane magnetic field $\mu_0 H_\parallel$ for Junction R, measured at 2 K over the field range −0.2 T ≤ $\mu_0 H_\parallel$ ≤ 0.2 T. Bottom panel shows the diode efficiency η, extracted from the corresponding critical currents (I_c^+, I_c^-) as a function of $\mu_0 H_\parallel$. (b) Corresponding measurements for Junction D under the same conditions, shown for direct comparison. (c) Corresponding measurements for Junction G under the same conditions, shown for direct comparison. (d) Diode efficiency η as a function of $|I_{avg}|$, extracted from the positive and negative critical currents (I_c^+, I_c^-) as a function of $\mu_0 H_\parallel$ for Junction R. (e) Corresponding measurements for Junction D under the same conditions, shown for direct comparison. (f) Corresponding measurements for Junction G under the same conditions, shown for direct comparison.

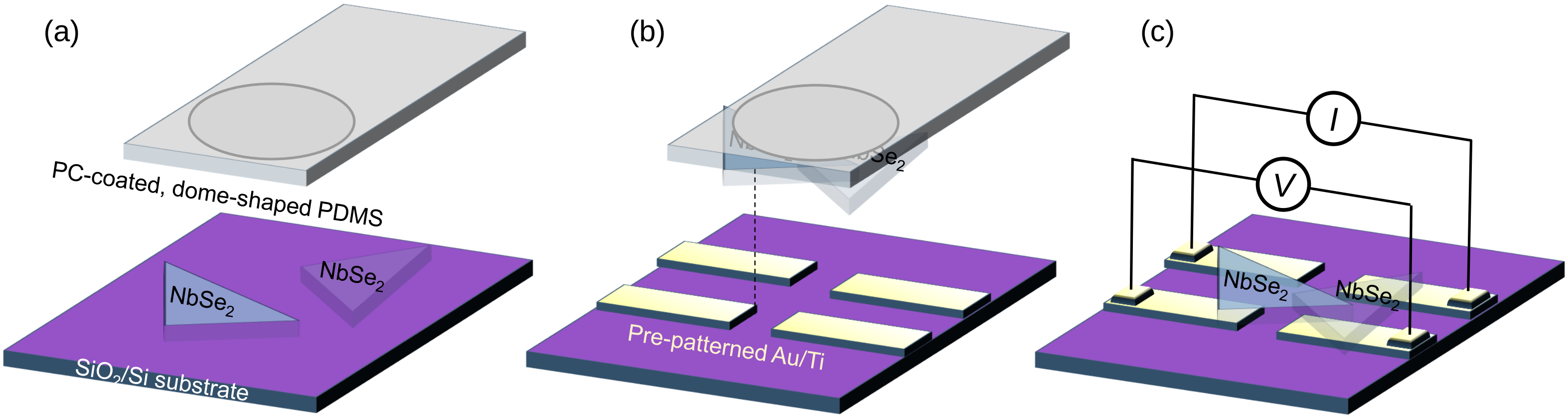
(a)
PC-coated, dome-shaped PDMS
NbSe2
NbSe2
SiO2/Si substrate
(b)
NbSe2
Pre-patterned Au/Ti
(c)
I
V
NbSe2
NbSe2

Fig. S1

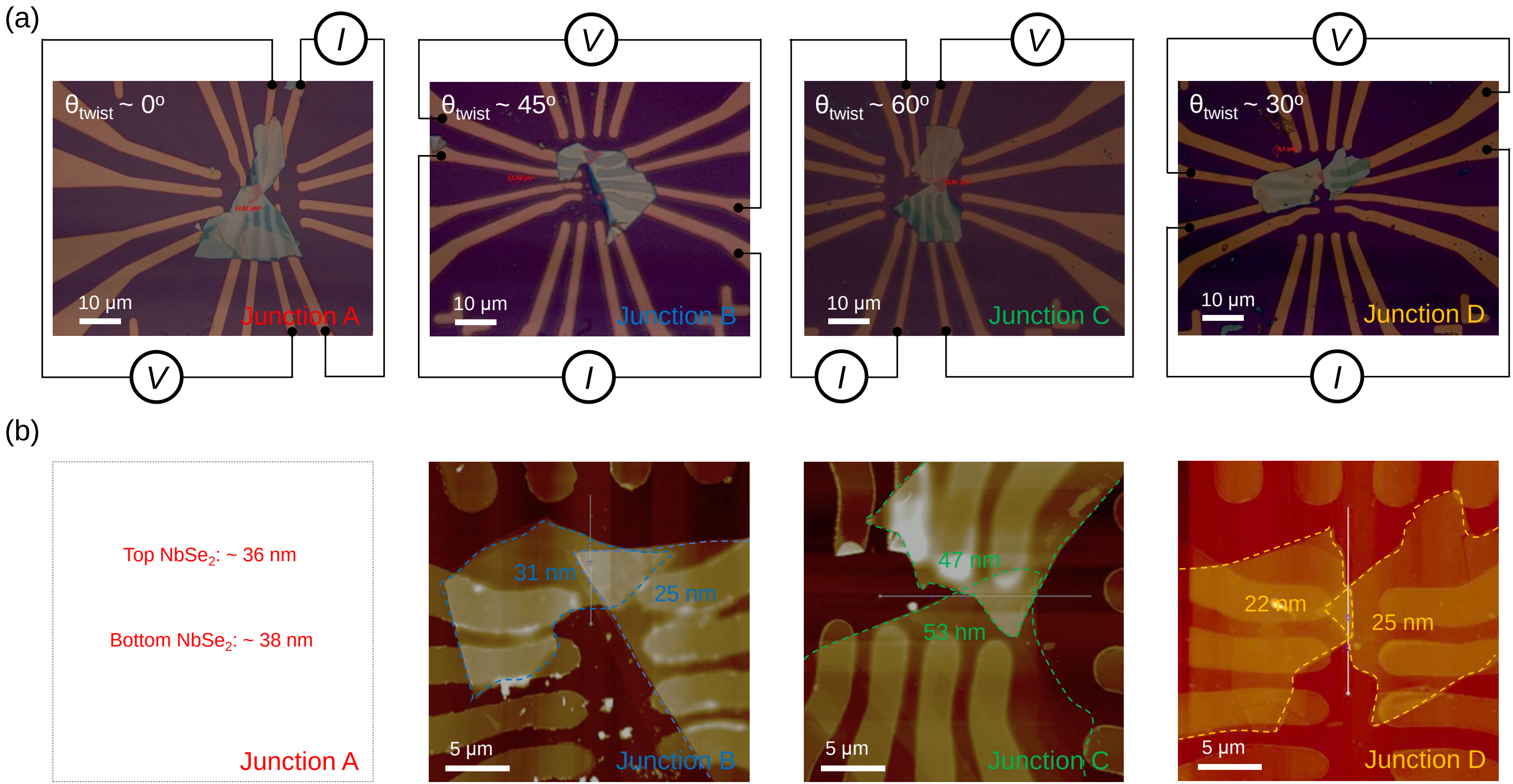
(a)
θtwist ~ 0º
10 μm
Junction A
I
V
θtwist ~ 45º
10 μm
Junction B
V
I
θtwist ~ 60º
10 μm
Junction C
V
I
θtwist ~ 30º
10 μm
Junction D
V
I
(b)
Top NbSe2: ~ 36 nm
Bottom NbSe2: ~ 38 nm
Junction A
31 nm
25 nm
5 μm
Junction B
47 nm
53 nm
5 μm
Junction C
22 nm
25 nm
5 μm
Junction D

Fig. S2

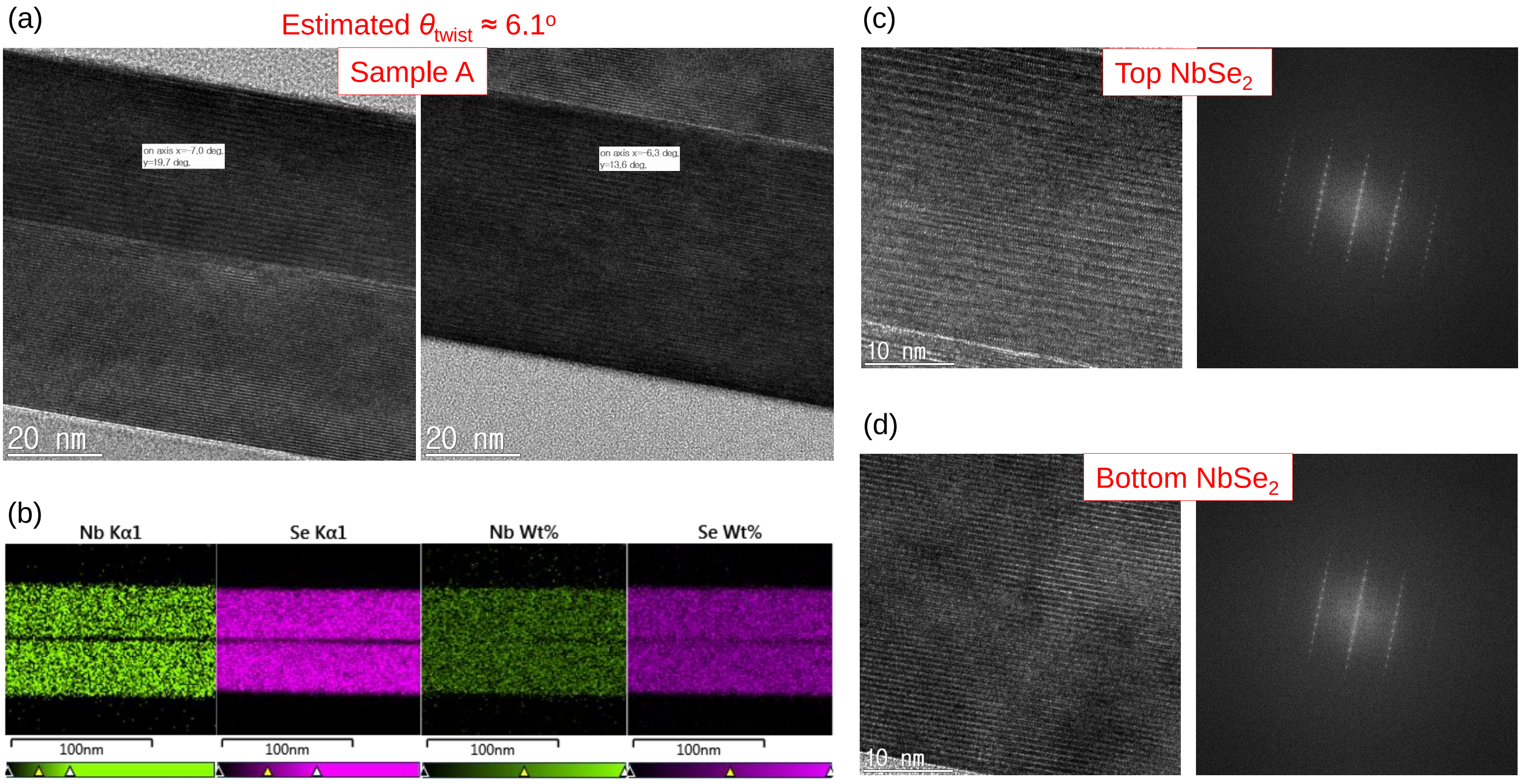
(a)
Estimated $\theta_{twist} \approx 6.1^o$
Sample A
on axis x=-7.0 deg.
y=19.7 deg.
on axis x=-6.3 deg.
y=13.6 deg.
20 nm
20 nm
(b)
Nb Kα1
Se Kα1
Nb Wt%
Se Wt%
100nm
100nm
100nm
100nm
(c)
Top $NbSe_2$
10 nm
(d)
Bottom $NbSe_2$
10 nm

Fig. S3

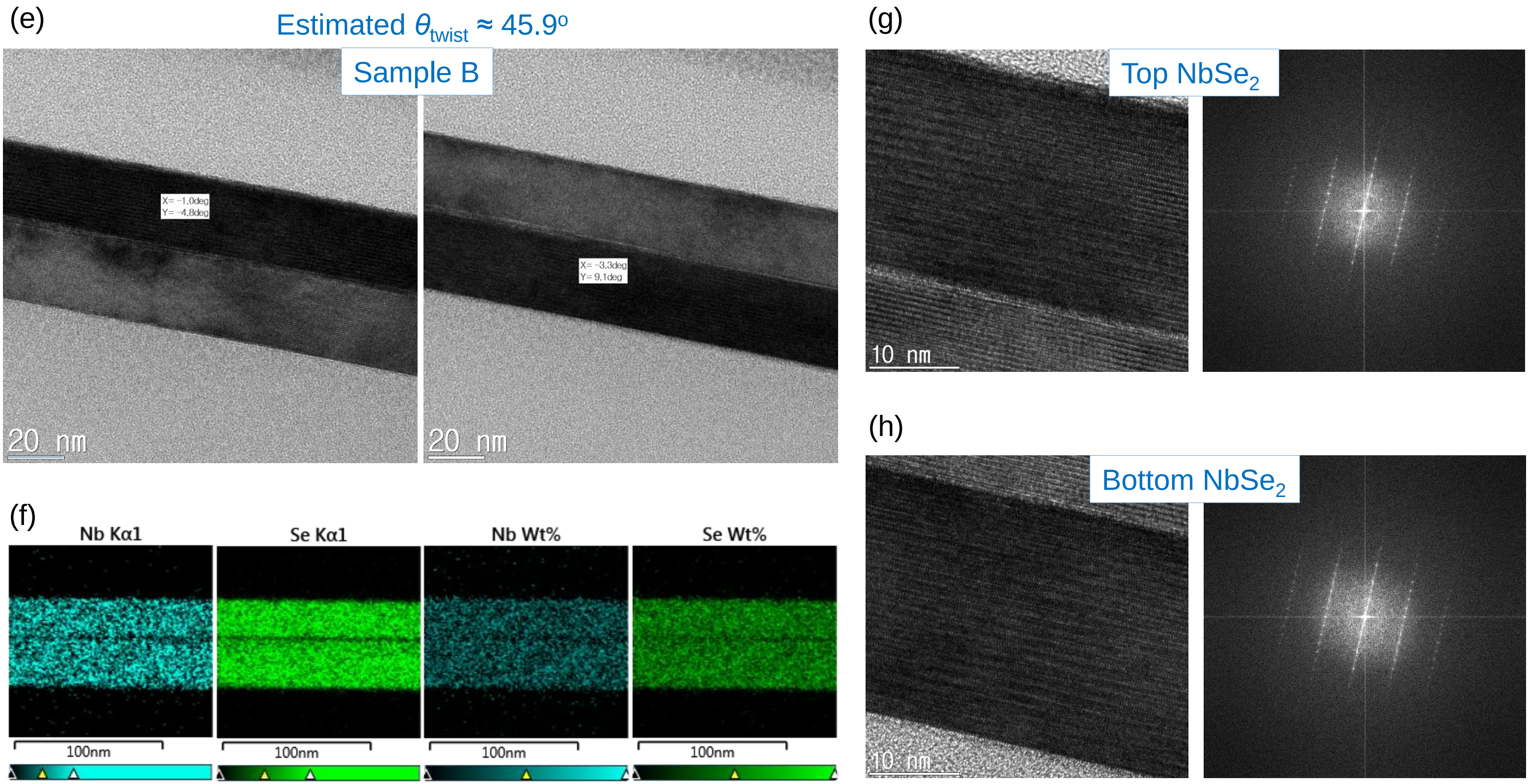

(e)
Estimated $\theta_{twist} \approx 45.9^o$
Sample B
X= -1.0deg
Y= -4.8deg
X= -3.3deg
Y= 9.1deg
20 nm
20 nm
(f)
Nb Kα1
Se Kα1
Nb Wt%
Se Wt%
100nm
100nm
100nm
100nm
(g)
Top $NbSe_2$
10 nm
(h)
Bottom $NbSe_2$
10 nm

Fig. S3

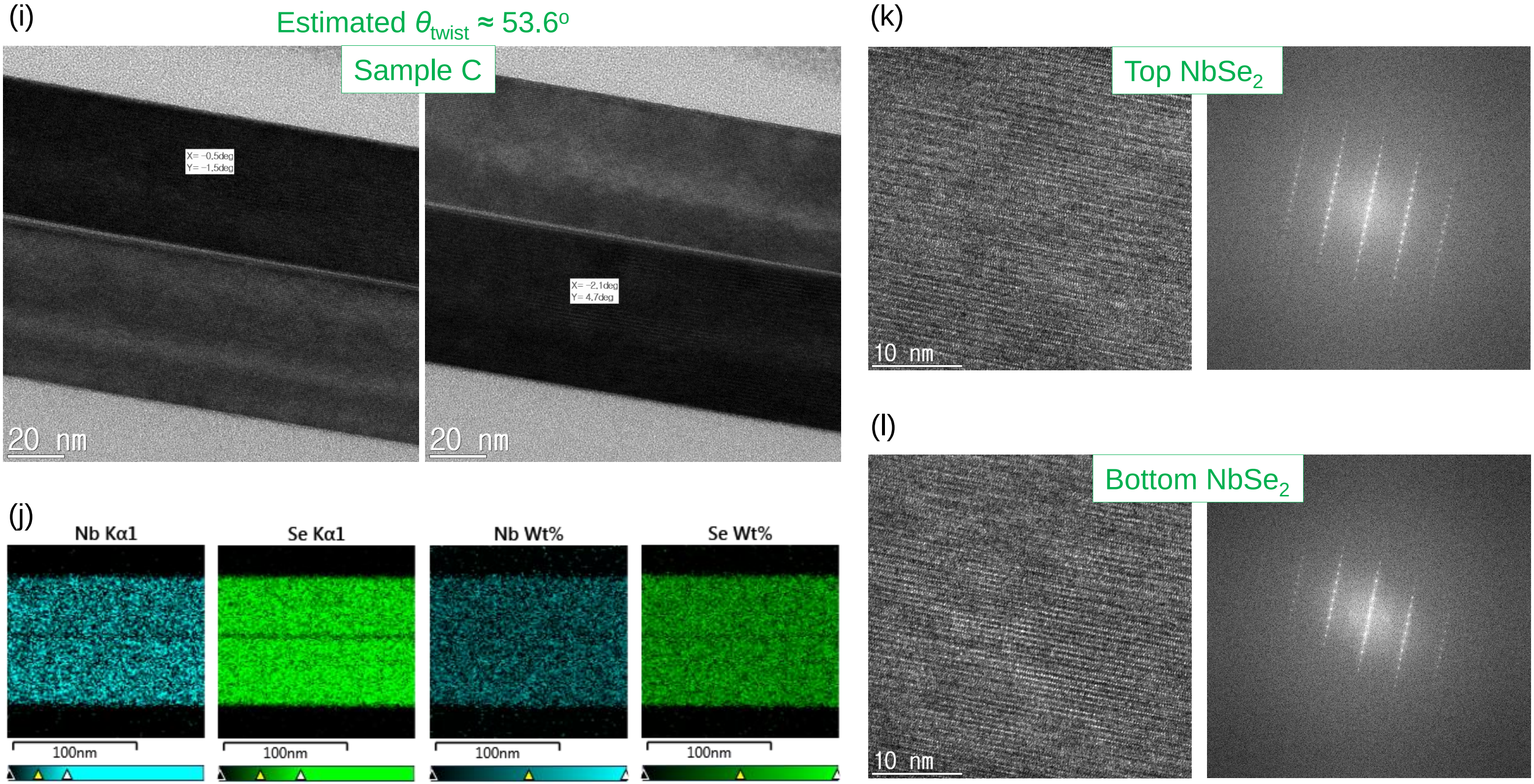
(i)
Estimated $\theta_{twist} \approx 53.6^o$
Sample C
X= −0.5deg
Y= −1.5deg
X= −2.1deg
Y= 4.7deg
20 nm
20 nm
(j)
Nb Kα1
Se Kα1
Nb Wt%
Se Wt%
100nm
100nm
100nm
100nm
(k)
Top $NbSe_2$
10 nm
(l)
Bottom $NbSe_2$
10 nm

Fig. S3

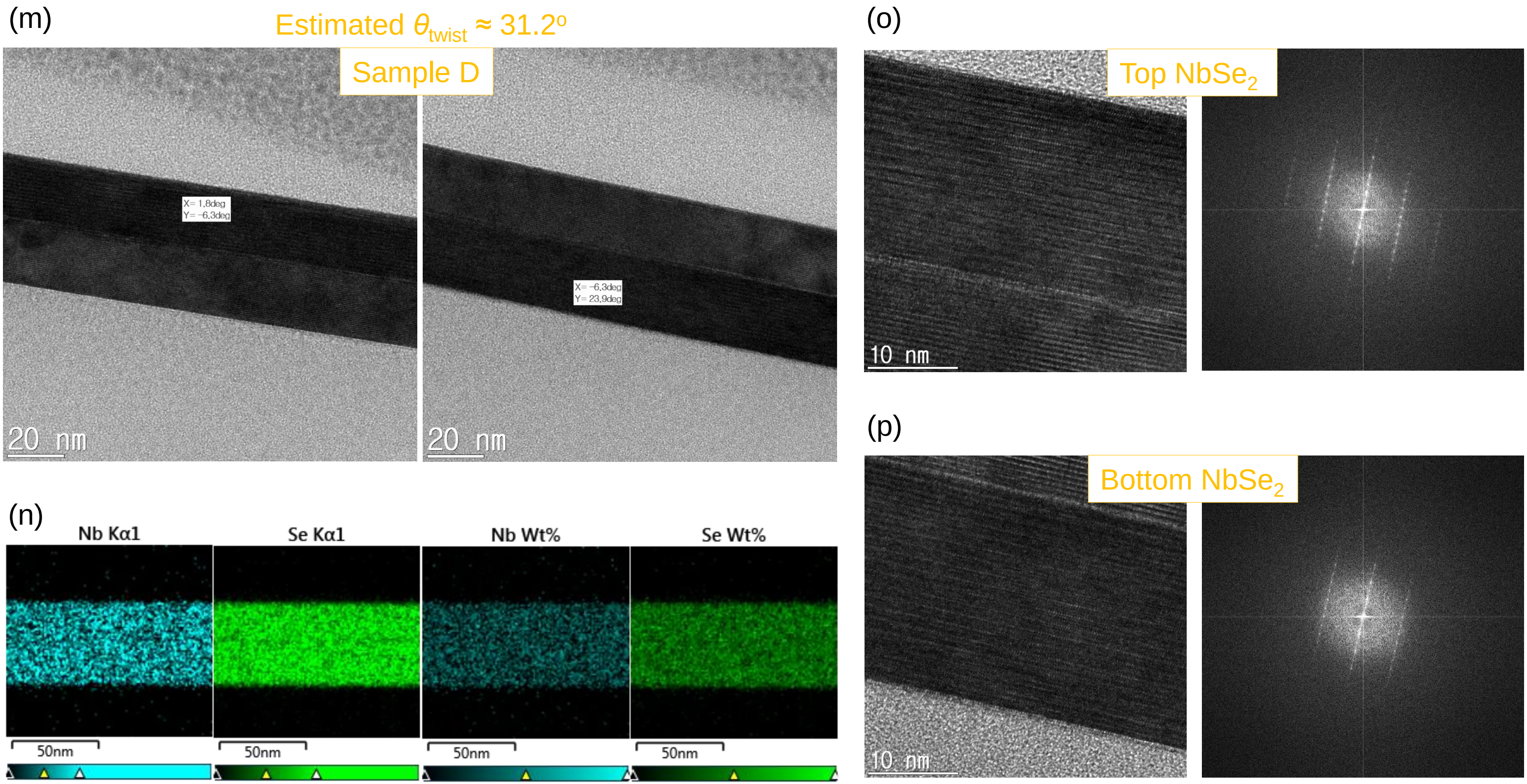

(m)
Estimated θtwist ≈ 31.2°
Sample D
X= 1.8deg
Y= -6.3deg
X= -6.3deg
Y= 23.9deg
20 nm
20 nm
(n)
Nb Kα1
Se Kα1
Nb Wt%
Se Wt%
50nm
50nm
50nm
50nm
(o)
Top NbSe2
10 nm
(p)
Bottom NbSe2
10 nm

Fig. S3

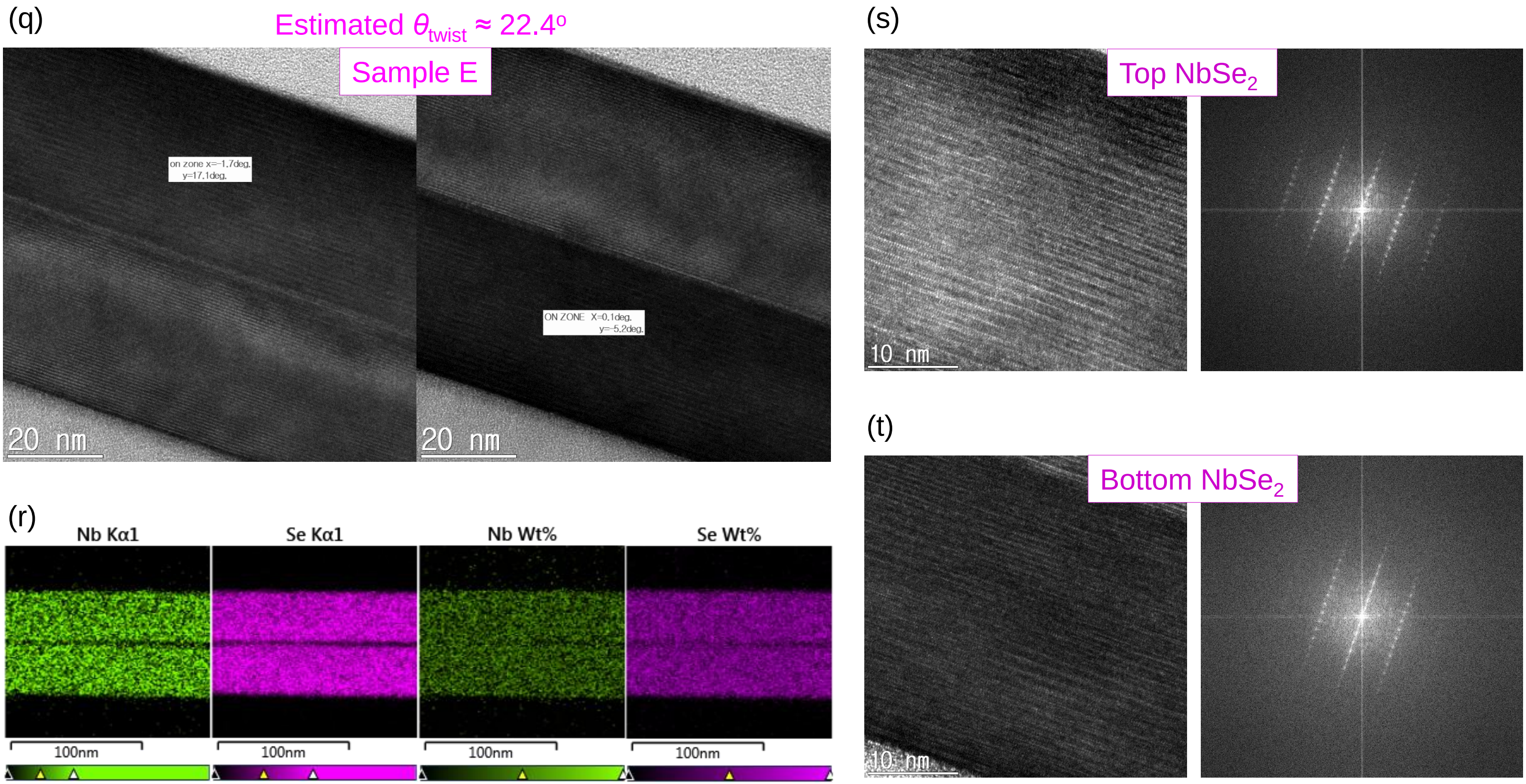
(q)
Estimated $\theta_{twist} \approx 22.4^o$
Sample E
on zone x=-1.7deg.
y=17.1deg.
ON ZONE X=0.1deg.
y=-5.2deg.
20 nm
20 nm
(r)
Nb Kα1
Se Kα1
Nb Wt%
Se Wt%
100nm
100nm
100nm
100nm
(s)
Top $NbSe_2$
10 nm
(t)
Bottom $NbSe_2$
10 nm

Fig. S3

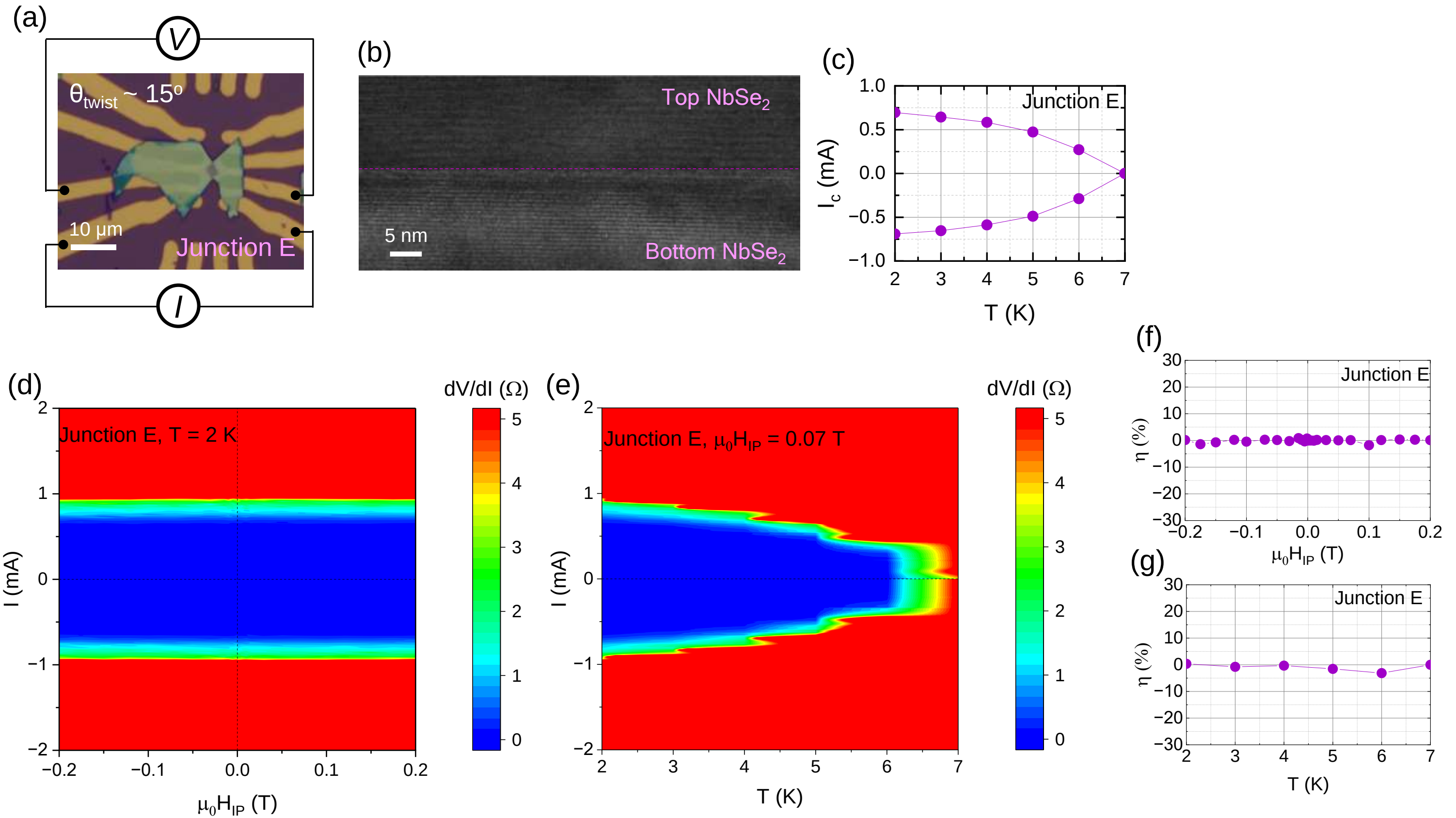

(a)
V
θtwist ~ 15°
10 μm
Junction E
I
(b)
Top NbSe2
5 nm
Bottom NbSe2
(c)
Junction E
Ic (mA)
T (K)
(d)
Junction E, T = 2 K
I (mA)
μ0HIP (T)
dV/dI (Ω)
(e)
Junction E, μ0HIP = 0.07 T
I (mA)
T (K)
dV/dI (Ω)
(f)
Junction E
η (%)
μ0HIP (T)
(g)
Junction E
η (%)
T (K)

Fig. S4

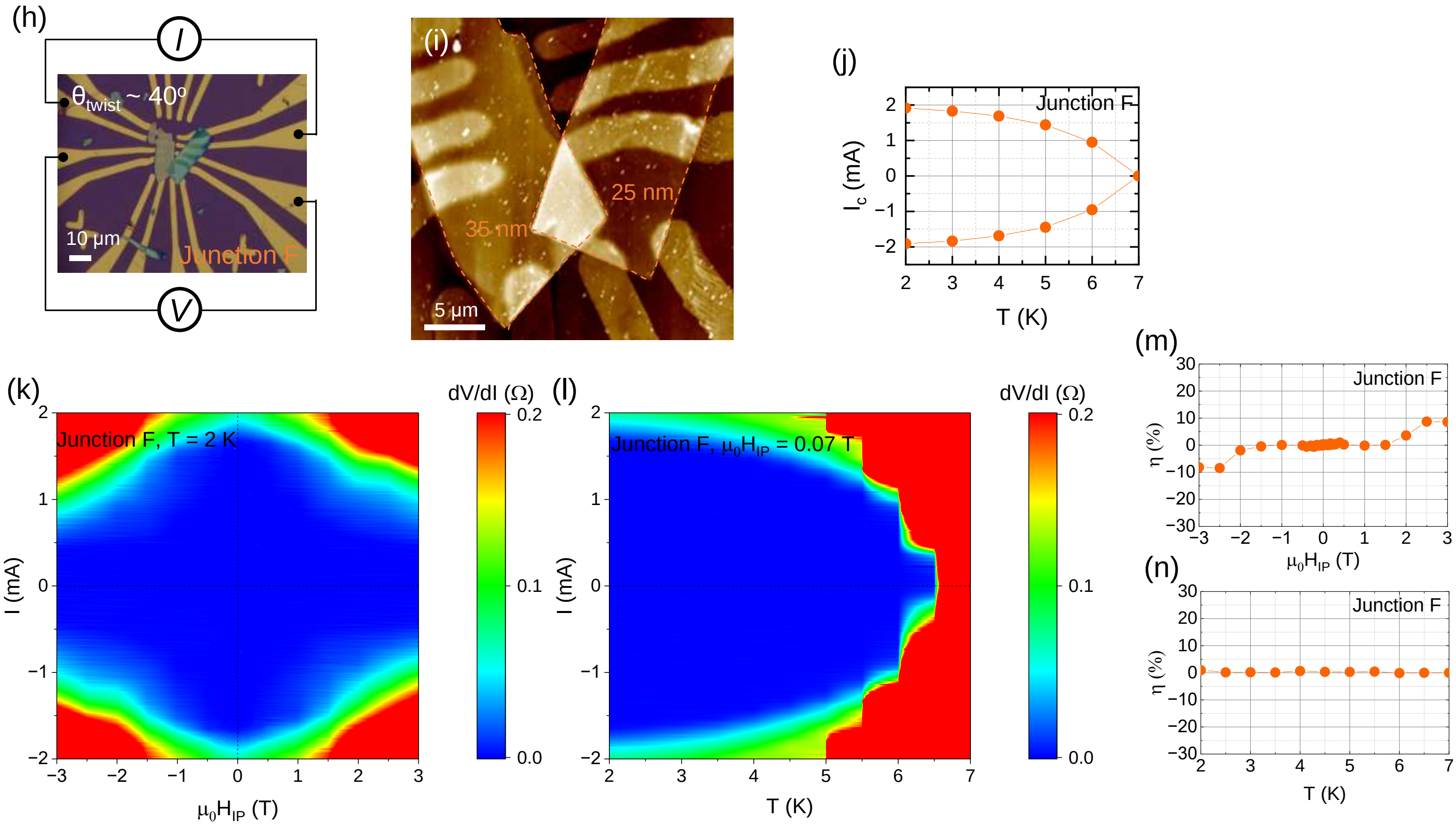
(h)
I
θtwist ~ 40º
10 µm
Junction F
V
(i)
35 nm
25 nm
5 µm
(j)
Junction F
Ic (mA)
T (K)
(k)
Junction F, T = 2 K
dV/dI (Ω)
I (mA)
µ0HIP (T)
(l)
Junction F, µ0HIP = 0.07 T
dV/dI (Ω)
I (mA)
T (K)
(m)
Junction F
η (%)
µ0HIP (T)
(n)
Junction F
η (%)
T (K)

Fig. S4

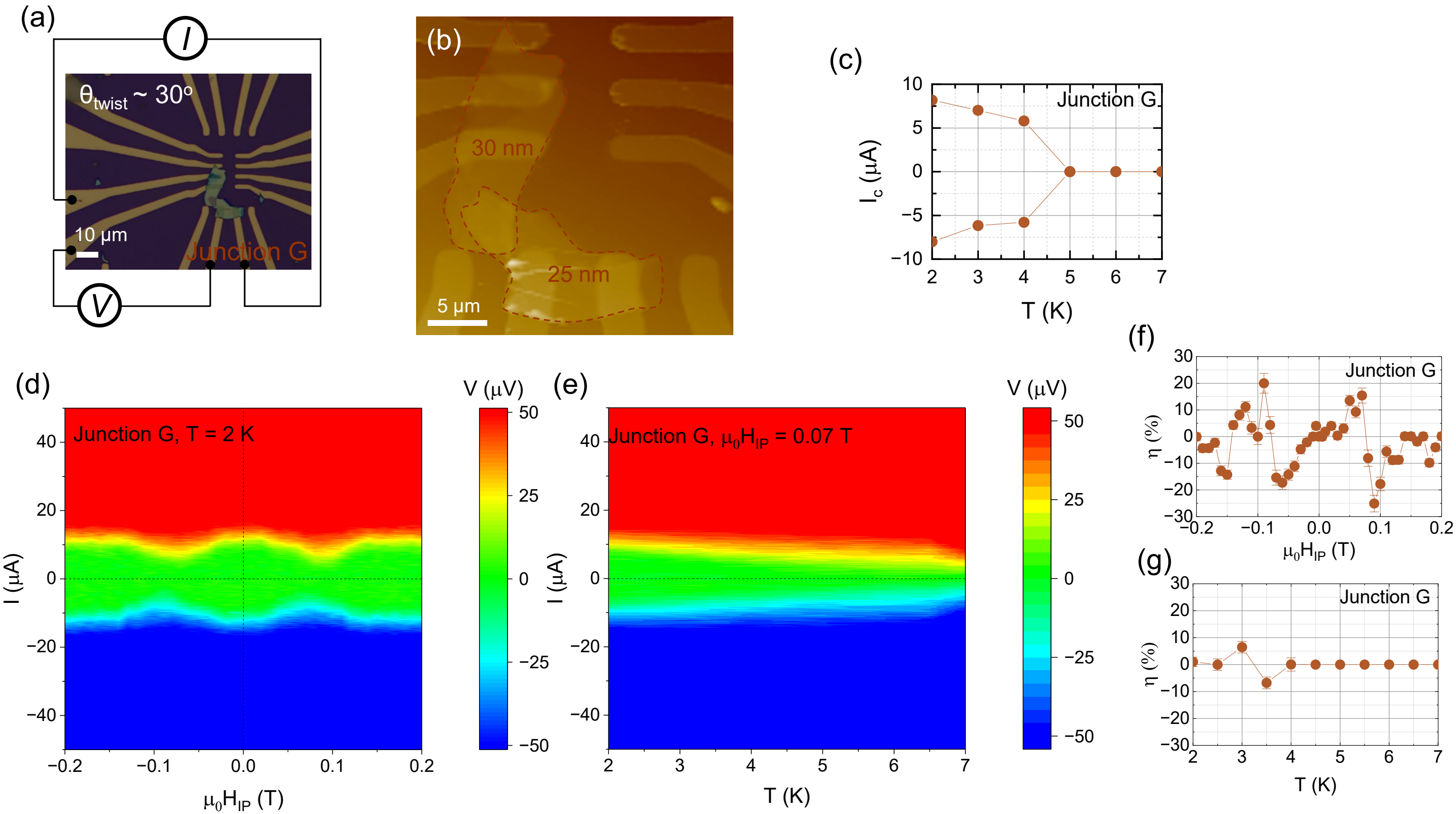
(a)
I
θtwist ~ 30°
10 μm
Junction G
V
(b)
30 nm
25 nm
5 μm
(c)
Junction G
Ic (μA)
T (K)
(d)
Junction G, T = 2 K
I (μA)
μ0HIP (T)
V (μV)
(e)
Junction G, μ0HIP = 0.07 T
I (μA)
T (K)
V (μV)
(f)
Junction G
η (%)
μ0HIP (T)
(g)
Junction G
η (%)
T (K)

Fig. S5

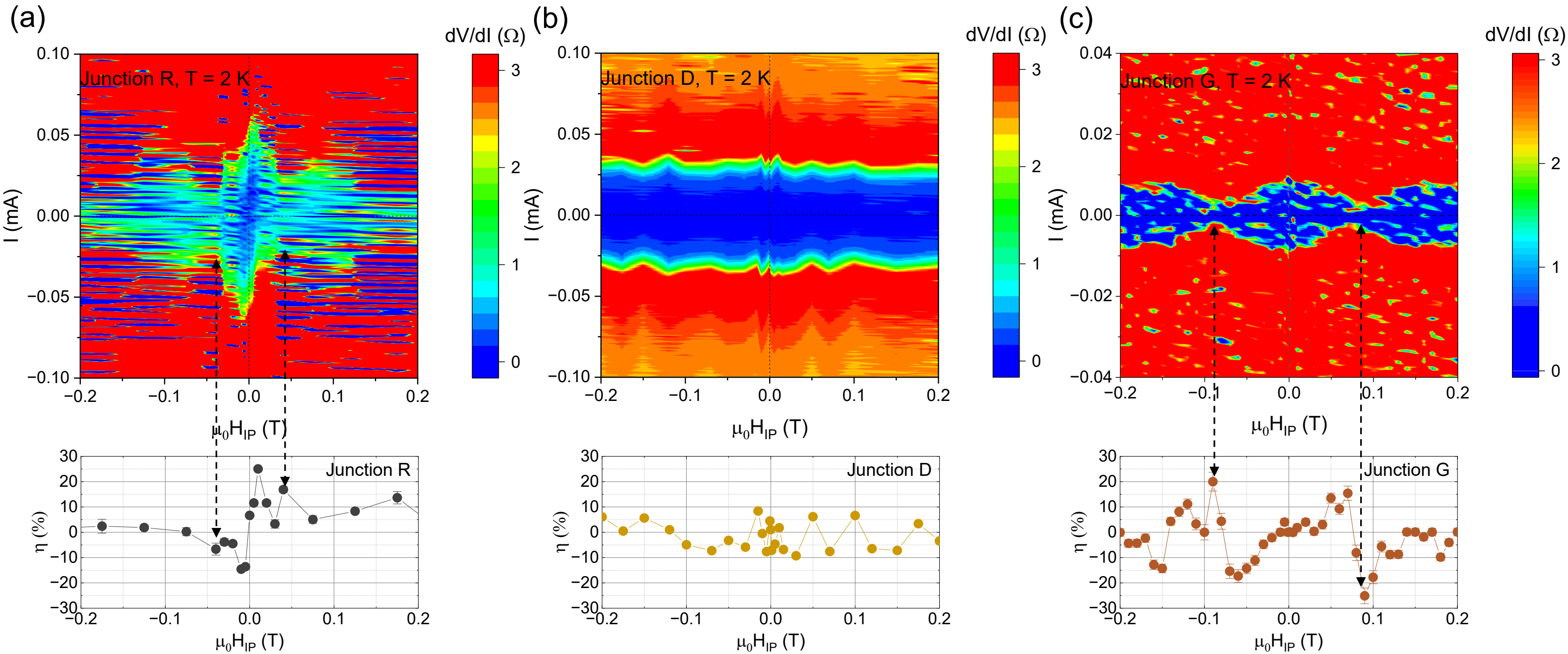

(a)
(b)
(c)
Junction R, T = 2 K
Junction D, T = 2 K
Junction G, T = 2 K
dV/dI (Ω)
I (mA)
μ0HIP (T)
η (%)
Junction R
Junction D
Junction G

Fig. S6

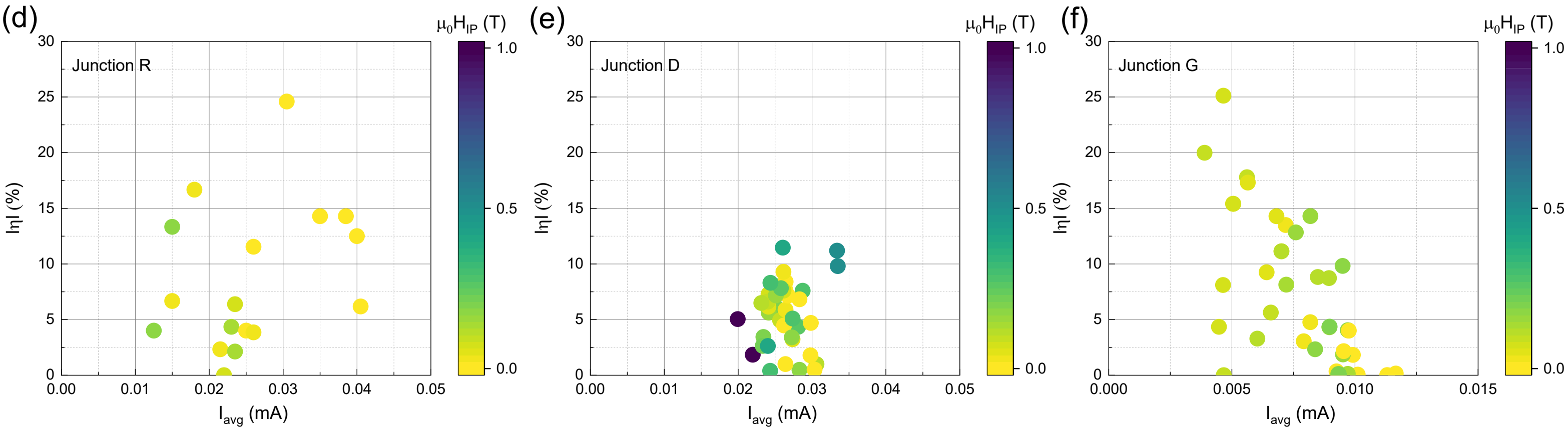
(d)
Junction R
|η| (%)
I_{avg} (mA)
$\mu_0 H_{IP}$ (T)
(e)
Junction D
|η| (%)
I_{avg} (mA)
$\mu_0 H_{IP}$ (T)
(f)
Junction G
|η| (%)
I_{avg} (mA)
$\mu_0 H_{IP}$ (T)

Fig. S6
